# Supplementary material for: Light-field flow cytometry for high-resolution, volumetric and multiparametric 3D single-cell analysis
Source: Nat Commun. 2024 Mar 4;15:1975. doi: 10.1038/s41467-024-46250-7 (PMC10912605; doi:10.1038/s41467-024-46250-7)
Supplement: Supplementary file 1 — Supplementary Information [file 41467_2024_46250_MOESM1_ESM.pdf]

SUPPLEMENTARY INFORMATION

Light-field flow cytometry for high-resolution, volumetric and multiparametric 3D single-cell analysis

Xuanwen Hua[1,†], Keyi Han[1,†], Biagio Mandracchia[1], Afsane Radmand[2,3], Wenhao Liu[1], Hyejin Kim[1], Zhou Yuan[2,4], Samuel M. Ehrlich[2,4], Kaitao Li[1], Corey Zheng[1], Jeonghwan Son[1], Aaron D. Silva Trenkle[1], Gabriel A. Kwong[1,2], Cheng Zhu[1,2], James E. Dahlman[1,2], and Shu Jia[1,2*]

[1]Wallace H. Coulter Department of Biomedical Engineering, Georgia Institute of Technology and Emory University, Atlanta, Georgia, USA
[2]Parker H. Petit Institute for Bioengineering and Biosciences, Georgia Institute of Technology, Atlanta, Georgia, USA
[3]Department of Chemical Engineering, Georgia Institute of Technology, Atlanta, Georgia, USA
[4]Georgia W. Woodruff School of Mechanical Engineering, Georgia Institute of Technology, Atlanta, Georgia, USA,
[†]These authors contributed equally: Xuanwen Hua, Keyi Han.

**E-mail: shu.jia@gatech.edu*

SUPPLEMENTARY INFORMATION

Table of Contents

Supplementary Figures **3**
Supplementary Note 1. Design of the microlens array (MLA) **16**
Supplementary Note 2. Flow focusing scheme **18**
Supplementary Note 3. Setup of stroboscopic illumination **19**
3.1. Using NI DAQ devices *20*
3.2. Using function-generating devices *21*
Supplementary Note 4. Vectorial Debye model **22**
Supplementary Note 5. Reconstruction algorithm using hybrid PSF **24**
Supplementary Note 6. Jurkat cell nucleus imaging with higher throughput **29**
Supplementary Note 7. Imaging system characterization **32**
7.1. Spatial resolution *32*
7.1.1. Lateral resolution *32*
7.1.2. Axial resolution *32*
7.2. Depth of focus (DOF) *33*
7.3. Field of view (FOV) *36*
7.4. Temporal resolution *36*
7.5. Analytical cell throughput *37*
7.6. Spatial resolution versus throughput *38*
Supplementary Note 8. Comparison with structured illumination microscopy for HeLa cells **40**
Supplementary Note 9. K-means clustering for blood cell morphology identification **45**
Supplementary Note 10. Comparison with structured illumination microscopy for Jurkat cells **46**
Supplementary Note 11. Comparison with the state-of-the-art IFC techniques **47**
Supplementary Note 12. System configurations for image processing **50**
Supplementary Note 13. Additional sample preparation **51**
13.1. Mouse platelet acquisition and staining *51*
13.2. Immunostaining for HeLa peroxisomes and mitochondria *52*
13.3. Floating Jurkat cell mounting onto the imaging dish *53*
Supplementary References **57**

Supplementary Figures

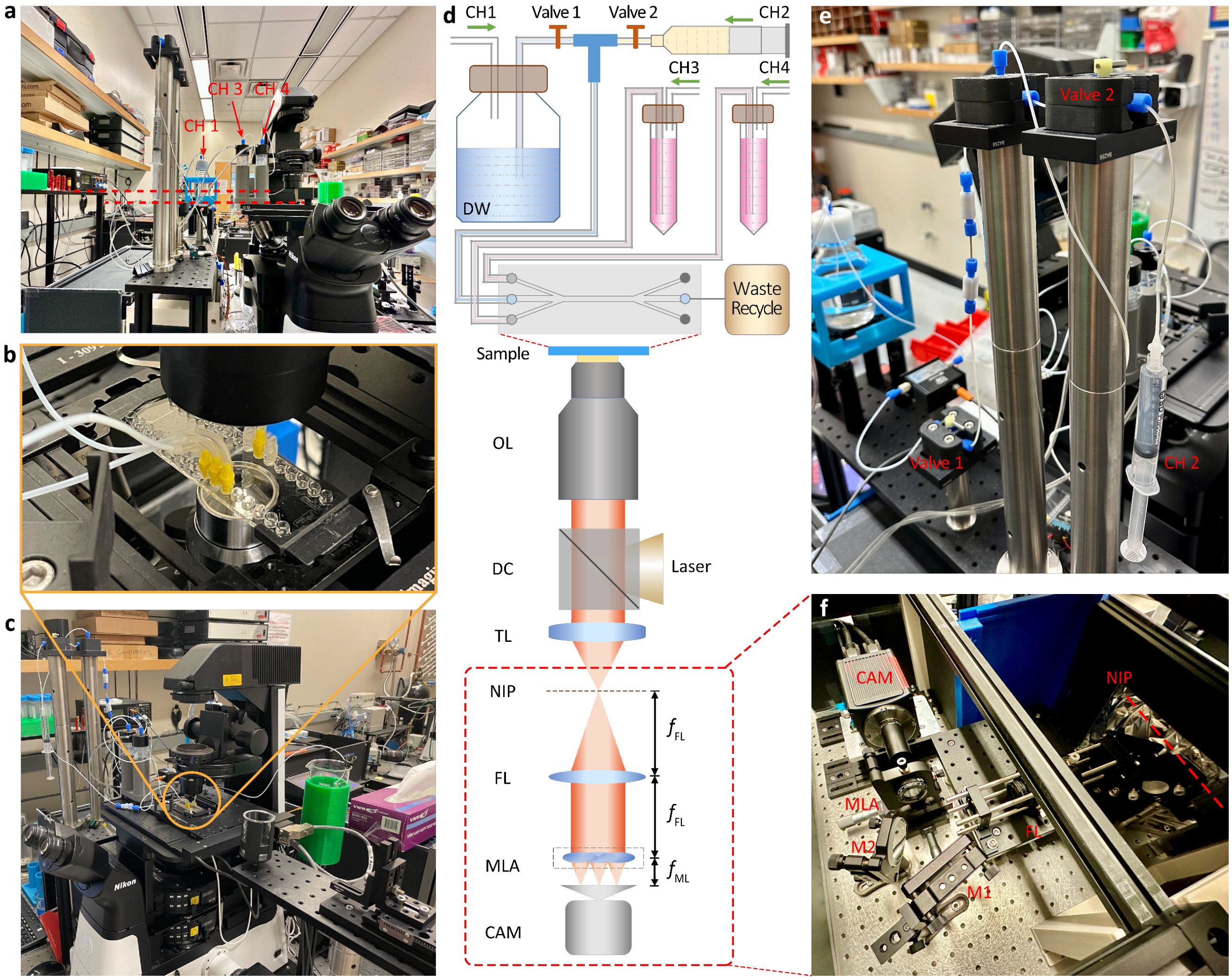

Supplementary Figure 1: Light-field flow cytometer (LFC). (a) Front view of LFC. The pump is slightly higher than CH1, 3, and 4 containers for stable control of microfluidics flow. (b) Close view of microfluidics chip loaded on the microscope with 3D-printed adapters. (c) Right view of LFC. (d) Schematic of LFC with detailed microfluidics illustration. CH1~4: Channel #1~4. DW: deionized water. OL: objective lens. DC: dichroic cube. TL: tube lens. NIP: native image plane. FL: Fourier lens. MLA: microlens array. CAM: camera. f_{FL}: focal length of Fourier lens. f_{ML}: focal length of each microlens. (e) Left view of the LFC. (f) Top view of Fourier light-field microscopy. M1, M2: mirrors.

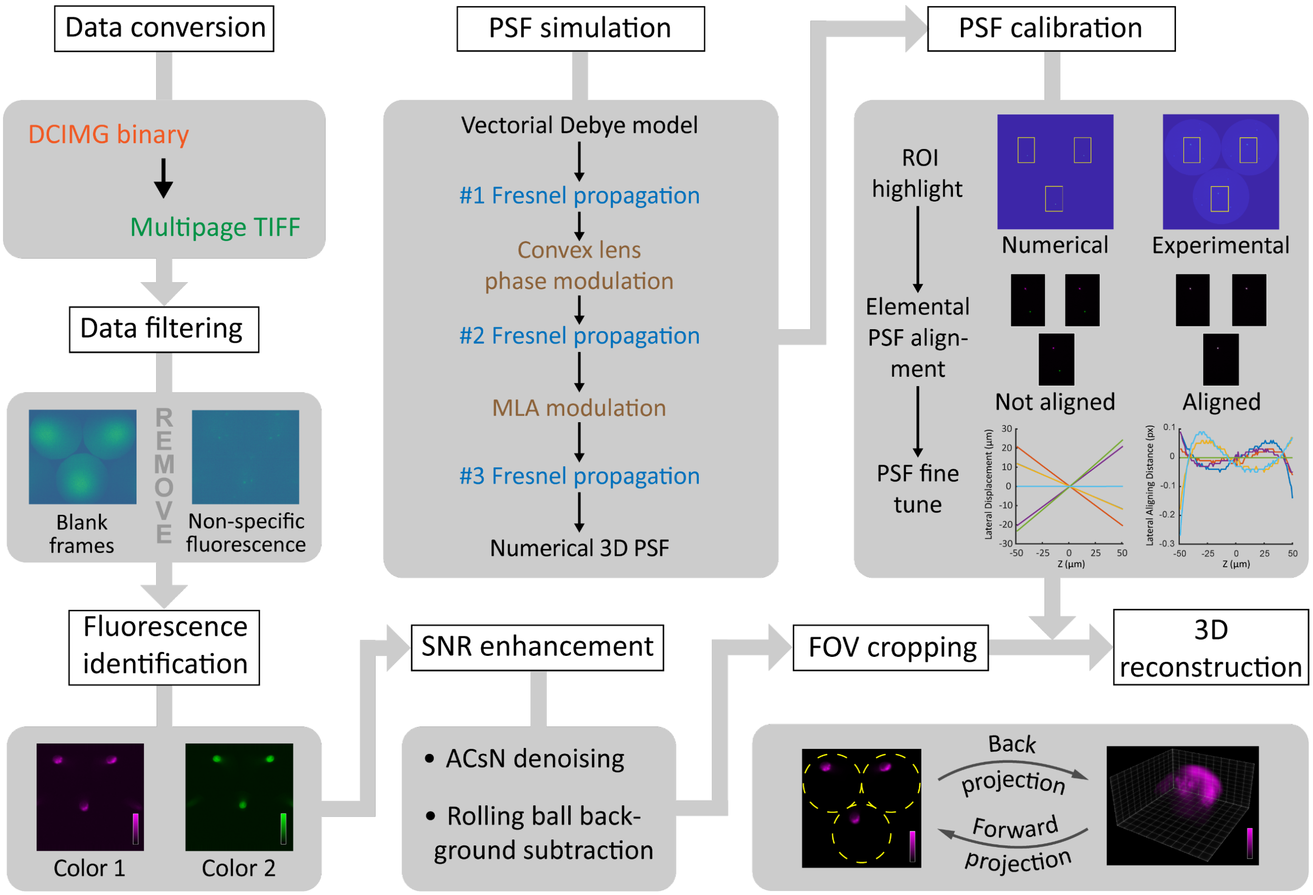

Supplementary Figure 2: Detailed data processing flowchart of the LFC. The raw data is first converted to "tiff" format. Then, the blank frames and frames with non-specific fluorescence are filtered out to increase the post-processing efficiency. The fluorescence types in the remaining frames are identified based on the organelle morphologies. Before reconstruction, the signal-to-noise ratio (SNR) is enhanced using both ACsN denoising[1] and rolling-ball background subtraction[2] methods. After denoising, the region outside field-of-view (FOV) in the three elemental images is removed to suppress the reconstruction artifacts. To perform better reconstruction, a hybrid point-spread-function (PSF) is generated with a simulated PSF and a PSF calibration scheme (Supplementary Software 1 and Supplementary Note 5). During the reconstruction, the volumetric information can be retrieved in an iterative manner with back projections and forward projections.

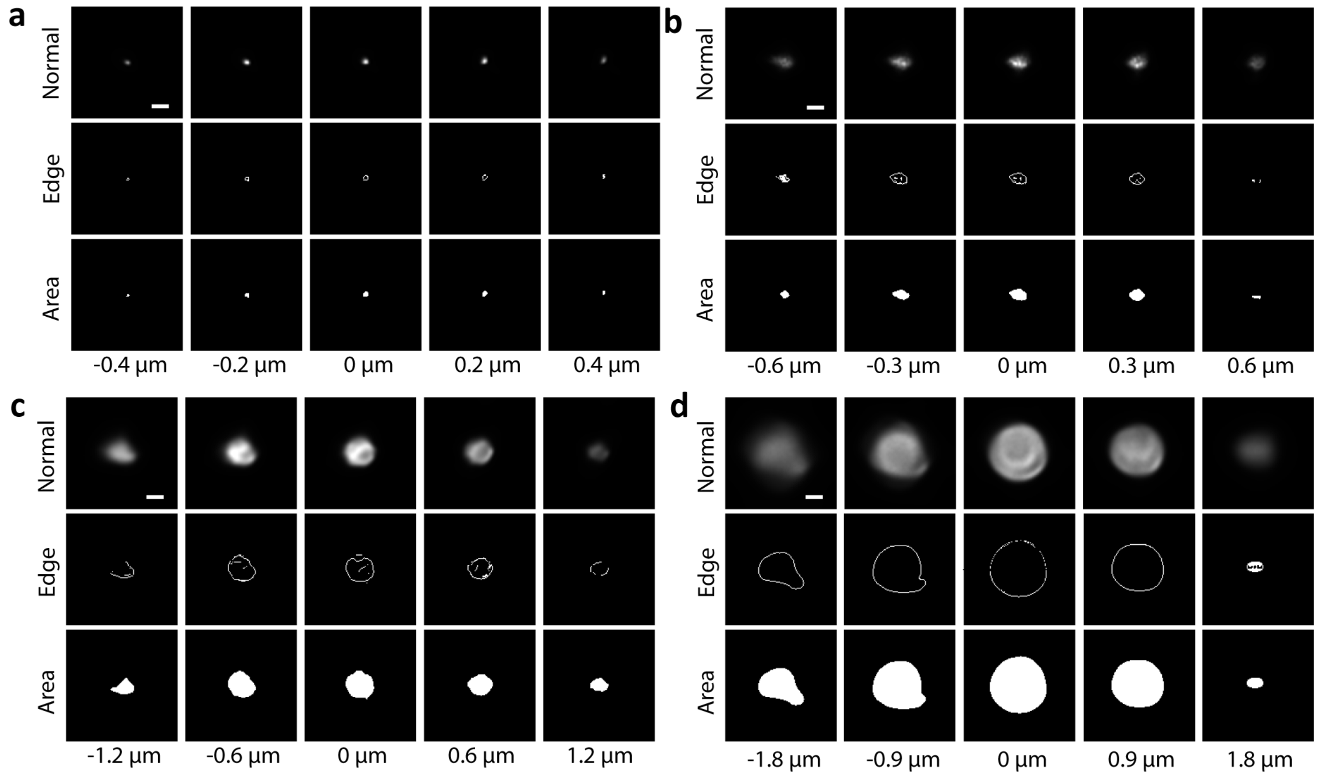

Supplementary Figure 3: Bead volume estimation scheme. (a) 200 nm beads. (b) 1 µm beads. (c) 2 µm beads. (d) 4 µm beads. In each panel, three rows are displayed. First row: 3D reconstructed images shown by layers. Second row: edge detection for each layer. Last row: Area calculation for all layers to estimate the 3D volume. Scale bars: 1 µm.

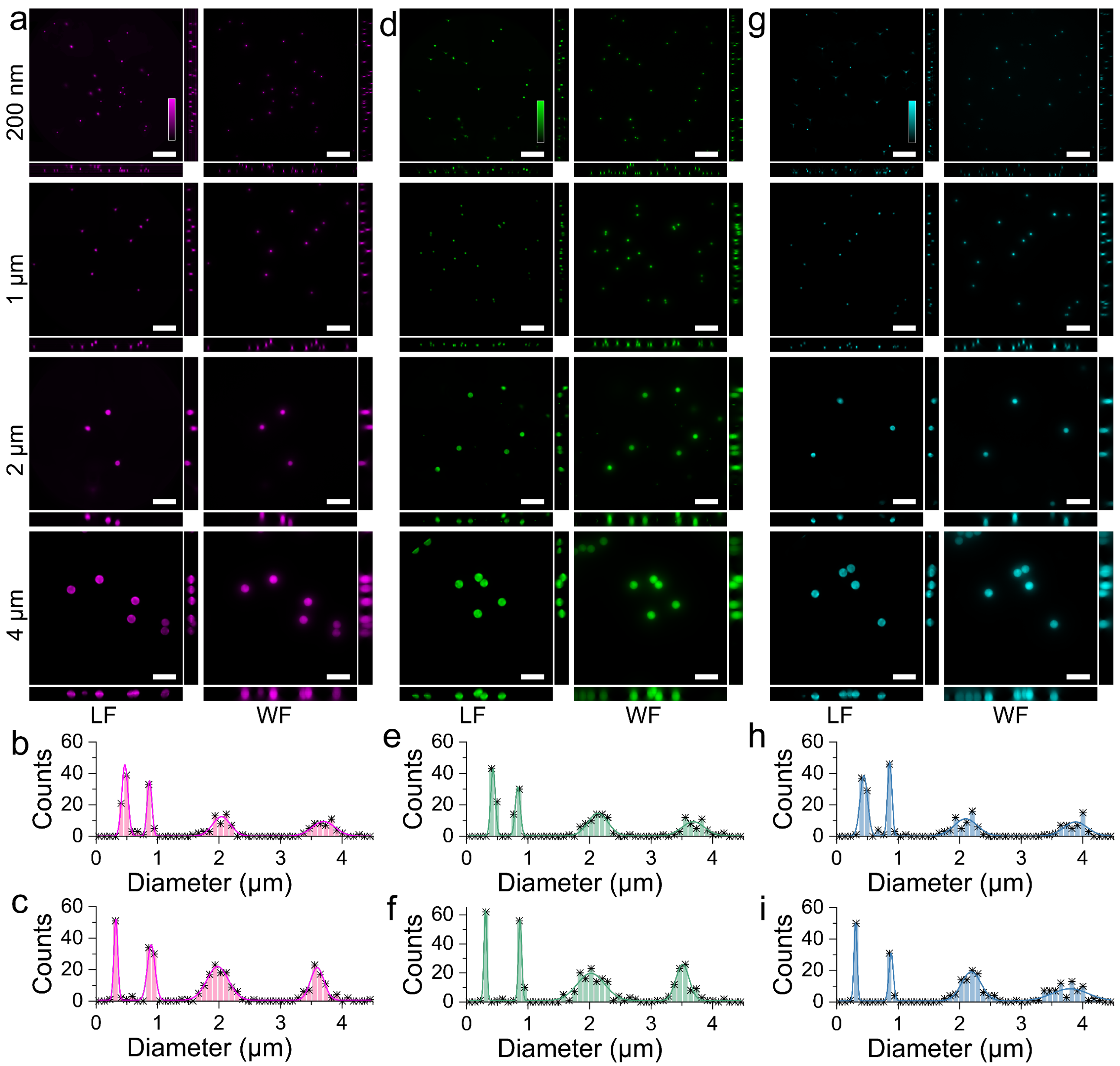

Supplementary Figure 4: Comparisons for bead volumes between wide-field imaging and light-field imaging. (a,d,g) Reconstructed light-field (LF) scanned volumes (left columns) and wide-field (WF) volumes (right columns) of 200-nm (top row), 1-µm (second row), 2-µm (third row), and 4-µm (bottom row) beads peaked at 680-nm (a), 599-nm (d), and 516-nm (g) fluorescent emission. (b,c,e,f,h,i) Histograms of bead diameters measured using light-field images (b,e,h) and wide-field images (c,f,i) of peak spectra at 680 nm (b, n = 377; c, n = 590), 599 nm (e, n = 312; f, n = 737), and 516 nm (h,n = 269; i, n = 543). Scale bars: 10 µm. Source data are provided as a Source Data file.

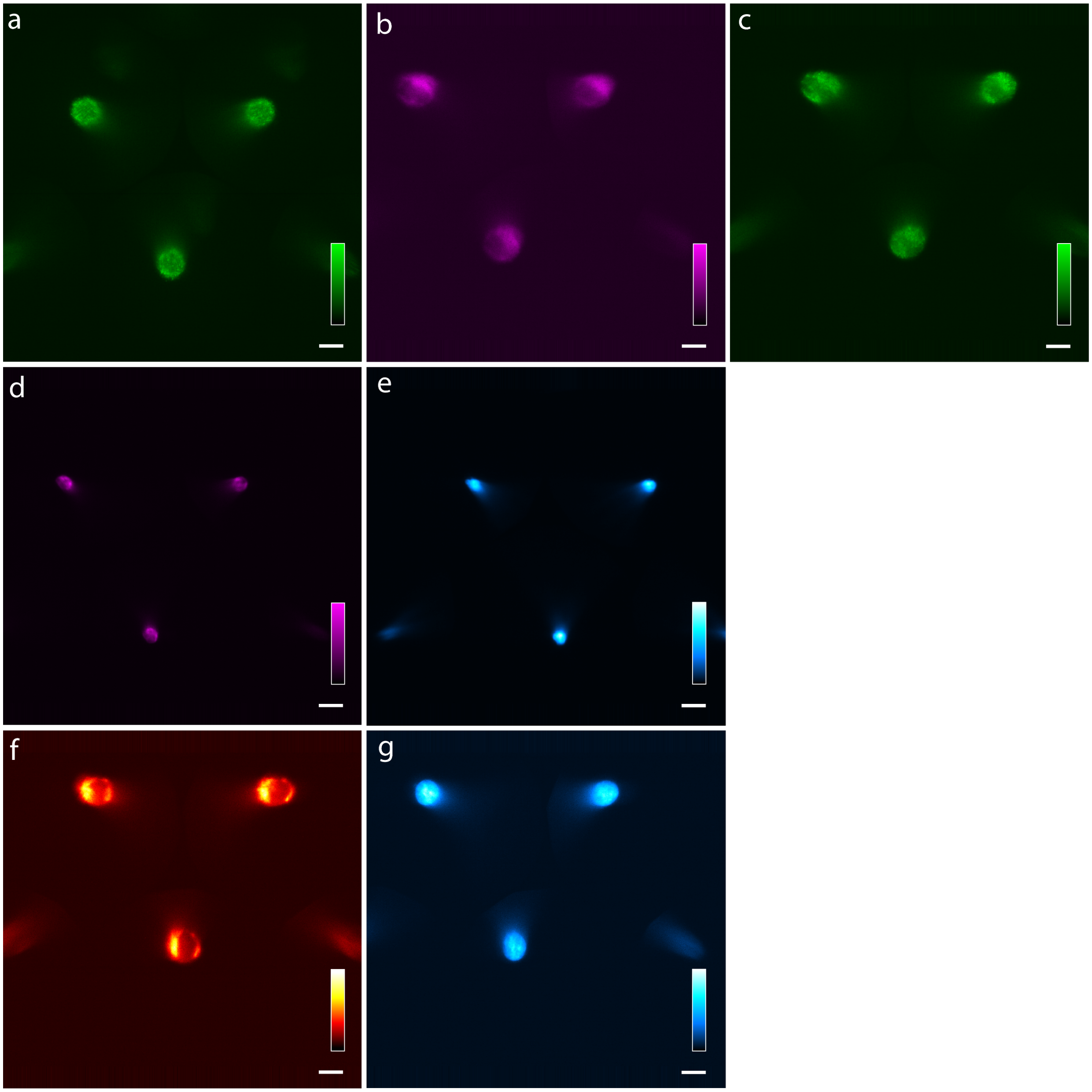

Supplementary Figure 5: Raw light-field images of Fig. 3-5. (a) Raw light-field image of Fig. 3a. (b) Raw light-field image of Fig. 3e. (c) Raw light-field image of Fig. 3f. (d, e) Raw light-field image of Fig. 4g. (f) Raw light-field image of Fig. 5a. (g) Raw light-field image of Fig. 5b. The pseudo-colors in the figures are consistent with those in the main figures. Scale bars: 10 μm.

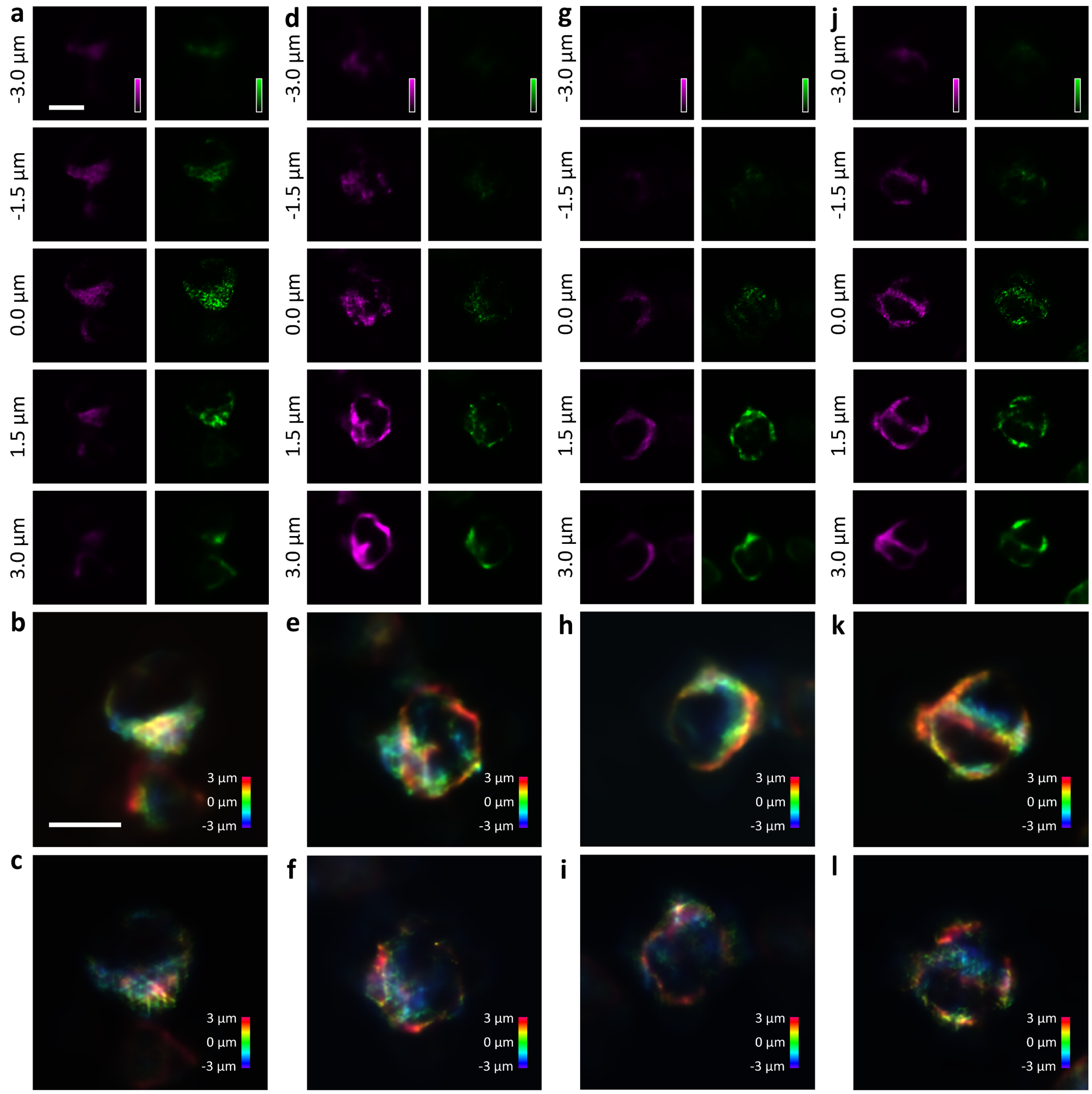

Supplementary Figure 6: Four examples of two-color imaging of mitochondria and peroxisome in flowing HeLa cells. (a, d, g, j) Two-color axial layers of 3D reconstructed HeLa cells in microfluidics flow from -3 µm to 3 µm. The magenta color is for mitochondria, and the green color is for peroxisomes. (b, e, h, k) Depth-color-coded reconstructed volume of HeLa mitochondria ranged from -3 µm to 3 µm. (c, f, i, l) Depth-color-coded reconstructed volume of HeLa peroxisomes ranged from -3 µm to 3 µm. Scale bars: 10 µm.

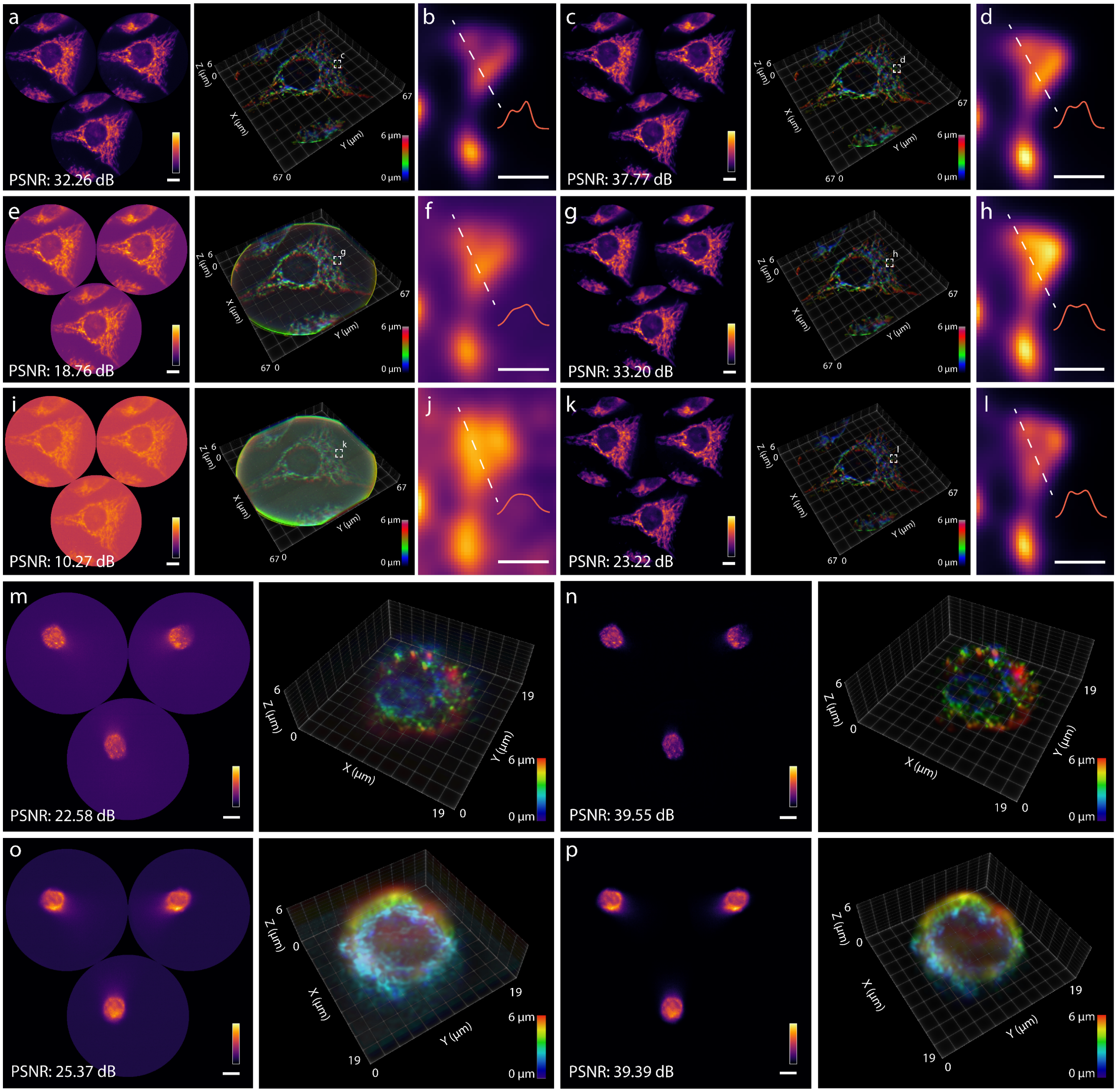

Supplementary Figure 7: Comparisons of reconstruction quality between light-field images with and without denoising algorithms at different noise levels. (a, e, i) Light-field images of HeLa mitochondria at different noise levels without denoising algorithms (left column) and their reconstructions (right column). (b, f, j) The zoomed-in region marked in the dashed boxes in (a), (e), and (i), respectively, with the intensity profiles along the dashed lines. (c, g, k) Light-field images of HeLa mitochondria at different noise levels with denoising algorithms (left column) and their reconstructions (right column). (d, h, l) The zoomed-in region marked in the dashed boxes in (c), (g), and (k), respectively, with the intensity profiles along the dashed lines. (m, o) Light-field images of HeLa peroxisomes and Jurkat mitochondria, respectively, at different noise levels without denoising algorithms (left column) and their reconstructions (right column). (n, p) Light-field images of HeLa peroxisomes and Jurkat mitochondria, respectively, at different noise levels with denoising algorithms (left column) and their reconstructions (right column). Scale bars: 10 μm (a, c, e, g, i, k, m, n, o, p), 1 μm (b, d, f, h, j, l). Source data are provided as a Source Data file.

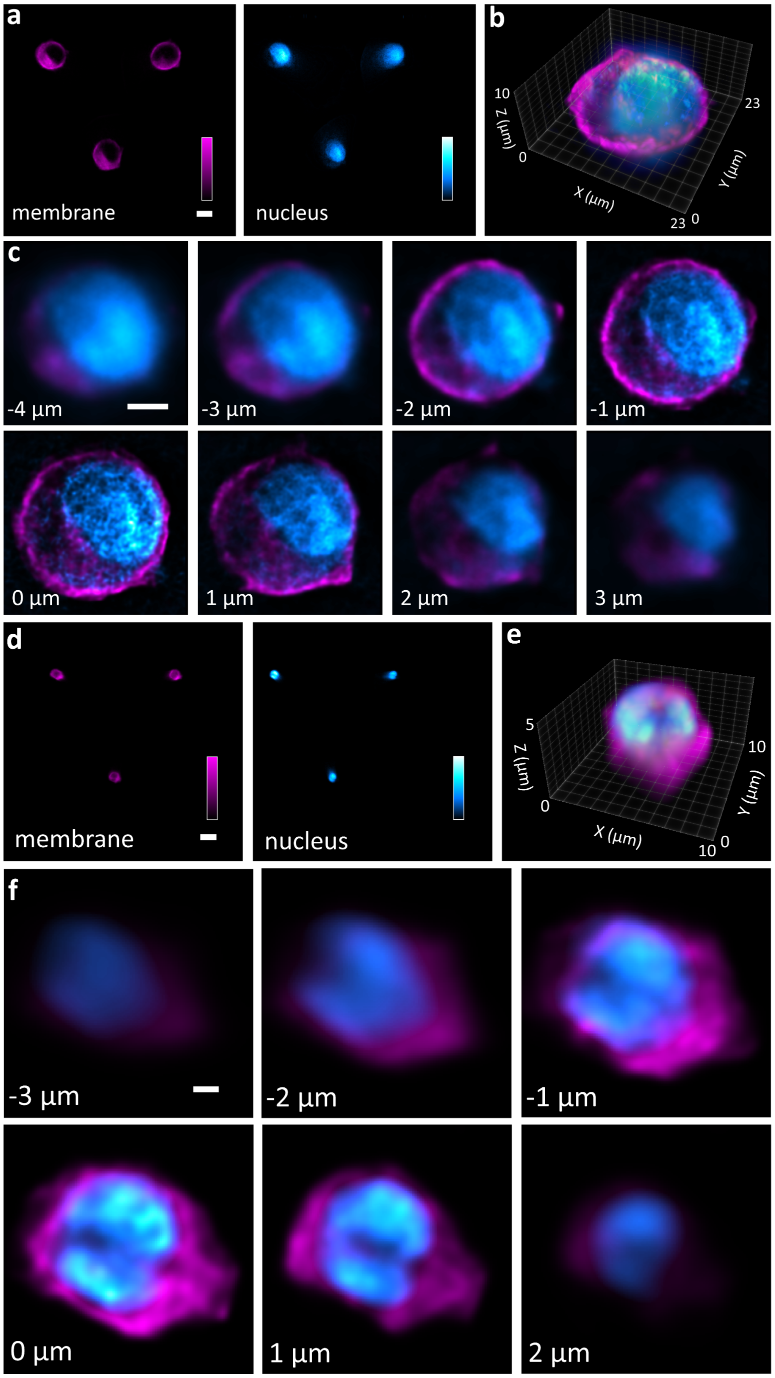

Supplementary Figure 8: Human HeLa cell and T cell nucleus and membrane imaging. (a) Light-field raw image of HeLa cell membrane (magenta) and nucleus (blue). (b) Visualization of 3D reconstructions of two structures in (a) with two-color integration. (c) Axial layers of the two-color volume in (b). (d) Light-field raw image of human T cell membrane (magenta) and nucleus (blue). (e) Visualization of 3D reconstructions of two structures in (d) with two-color integration. (f) Axial layers of the two-color volume in (e). Scale bars: 10 μm (a, d), 5 μm (c), 1 μm (f).

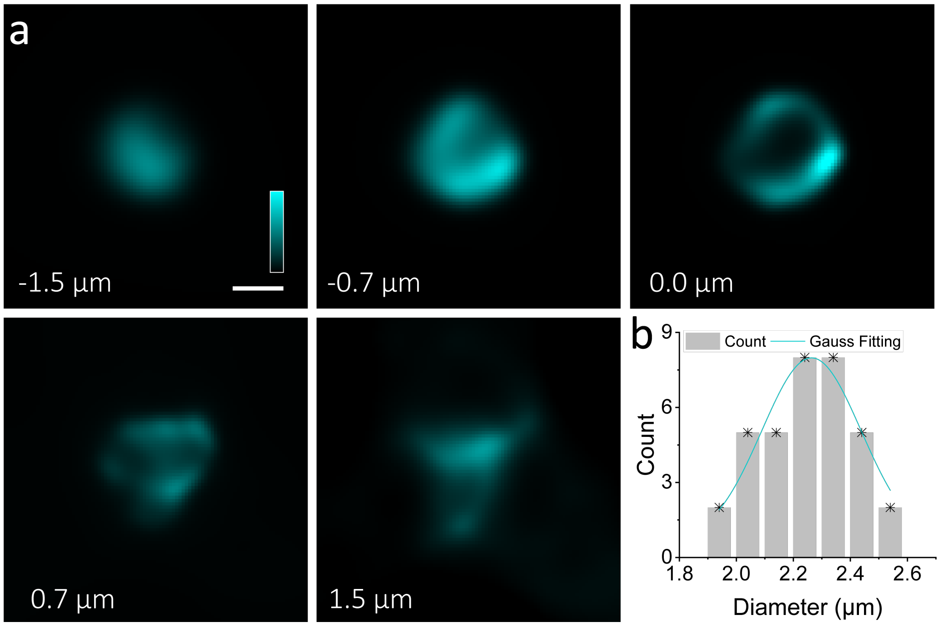

Supplementary Figure 9: Imaging platelets at a flow rate of ~2,300 cells/sec using LFC. (a) Axial layers for a single membrane-labeled platelet volume across 3 µm. (b) Histogram of the diameters of platelets (n = 35) with the assumption that the platelets are spherical. The Gaussian fitting of the histogram shows a mean diameter of 2.26 µm. Scale bar: 1 µm (a). Source data are provided as a Source Data file.

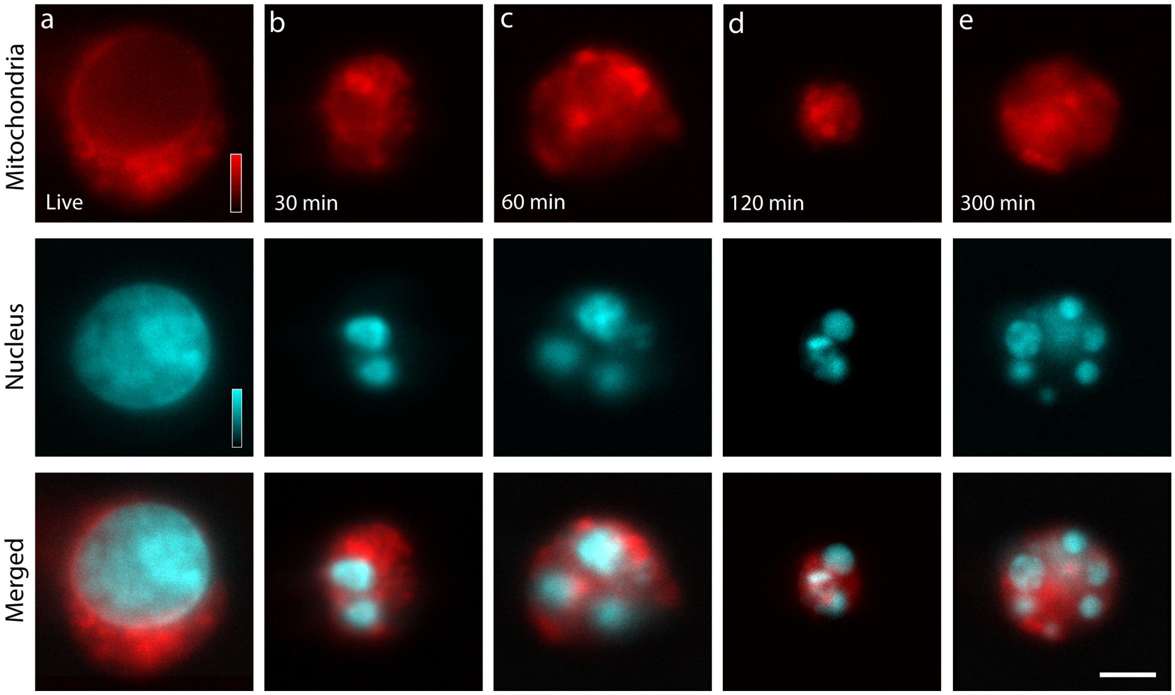

Supplementary Figure 10: Wide-field images of mitochondria and nuclei in both live Jurkat cells and STS-treated cells in microfluidics. (a) Live cells with no STS treatment. (b) 30 min STS treatment. (c) 60 min STS treatment. (d) 120 min STS treatment. (e) 300 min STS treatment. Scale bar: 5 μm.

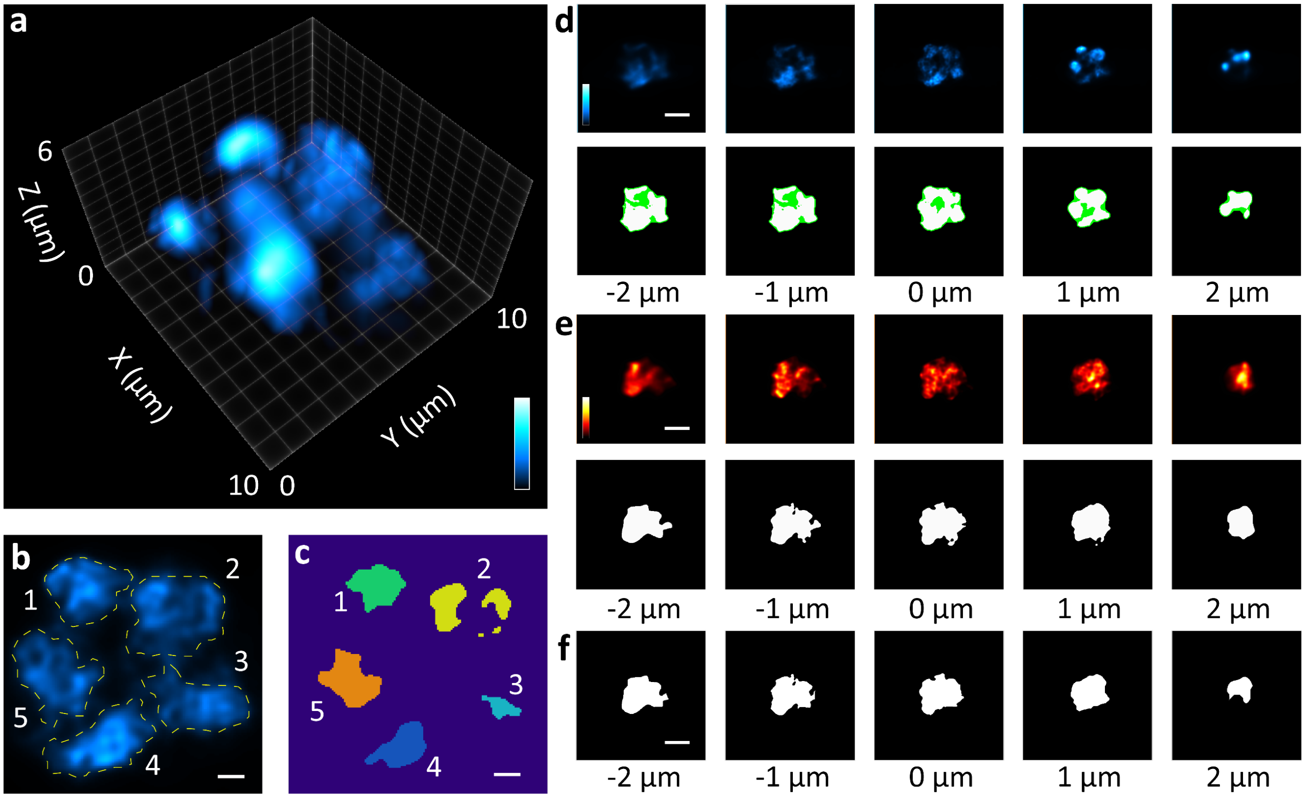

Supplementary Figure 11: Jurkat cell nucleus and mitochondria segmentation and volume estimation. (a) 3D visualization of the reconstructed volume of Jurkat cell nucleus. (b) One axial layer of (a) with dashed-area segmentation was manually marked for each broken nucleus part. (c) 3D nucleus segmentation was performed by algorithm, consistent with manual segmentation results. (d) Axial layers of nuclei 3D reconstruction (first row) and their enclosed areas (second row, white part represents nucleus while green part represents enclosed area). (e) Axial layers of mitochondria 3D reconstruction (first row) and their recognized areas (second row). (f) The intersection of the nucleus-enclosed regions in (d) and mitochondria areas in (e). Scale bars: 1 μm (b, c), 5 μm (d-f).

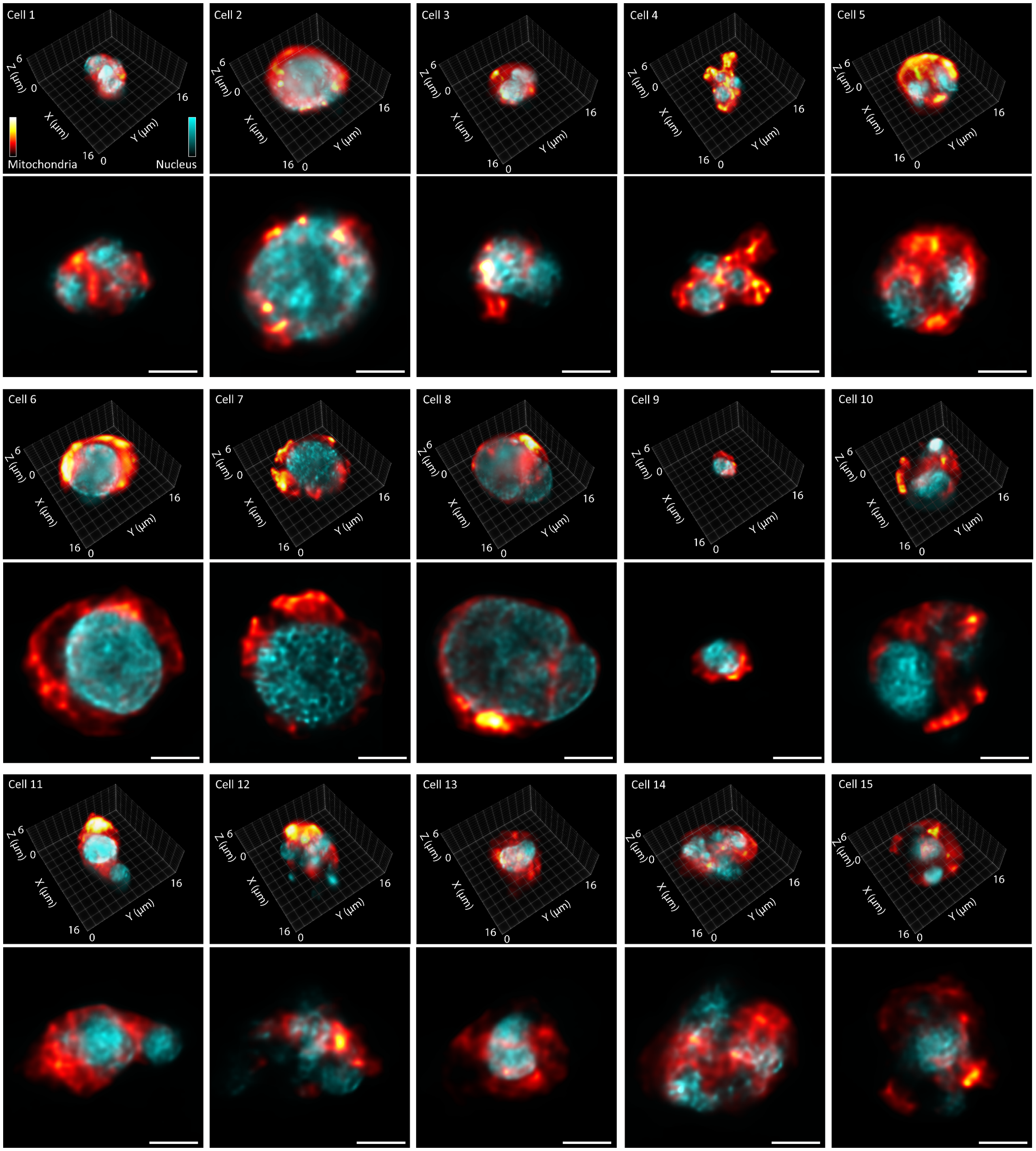

Supplementary Figure 12: Two-color imaging of mitochondria and nucleus in 15 exemplary flowing Jurkat T cells with 300-min STS treatment times. The three panels show 3D rendering on the top of each panel and corresponding focal stack images at the bottom of each panel. A majority of results displayed a consistent resolution of the subcellular structures compared to those of other treatment times in Fig. 5. Scale bars: 5 µm.

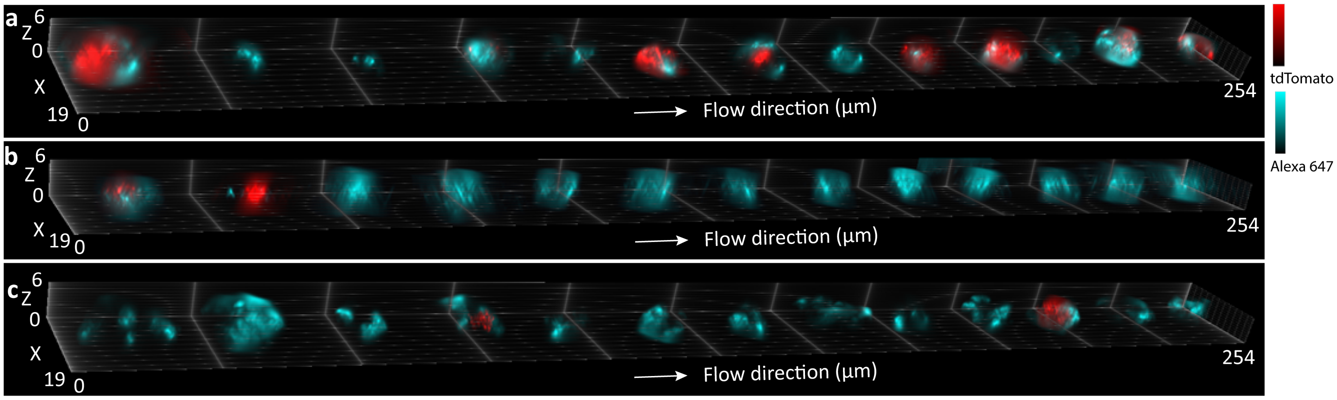

Supplementary Figure 13: Flow montage of liver endothelial cells, spleen immune cells, and lung endothelial cells with nanoparticles injected. (a) Mouse liver endothelial cells. (b) Mouse spleen immune cells. (c) Mouse lung endothelial cells. The cell membrane was displayed in cyan color, and tdTomato expression inside the cells was displayed in red color.

Supplementary Note 1. Design of the microlens array (MLA)

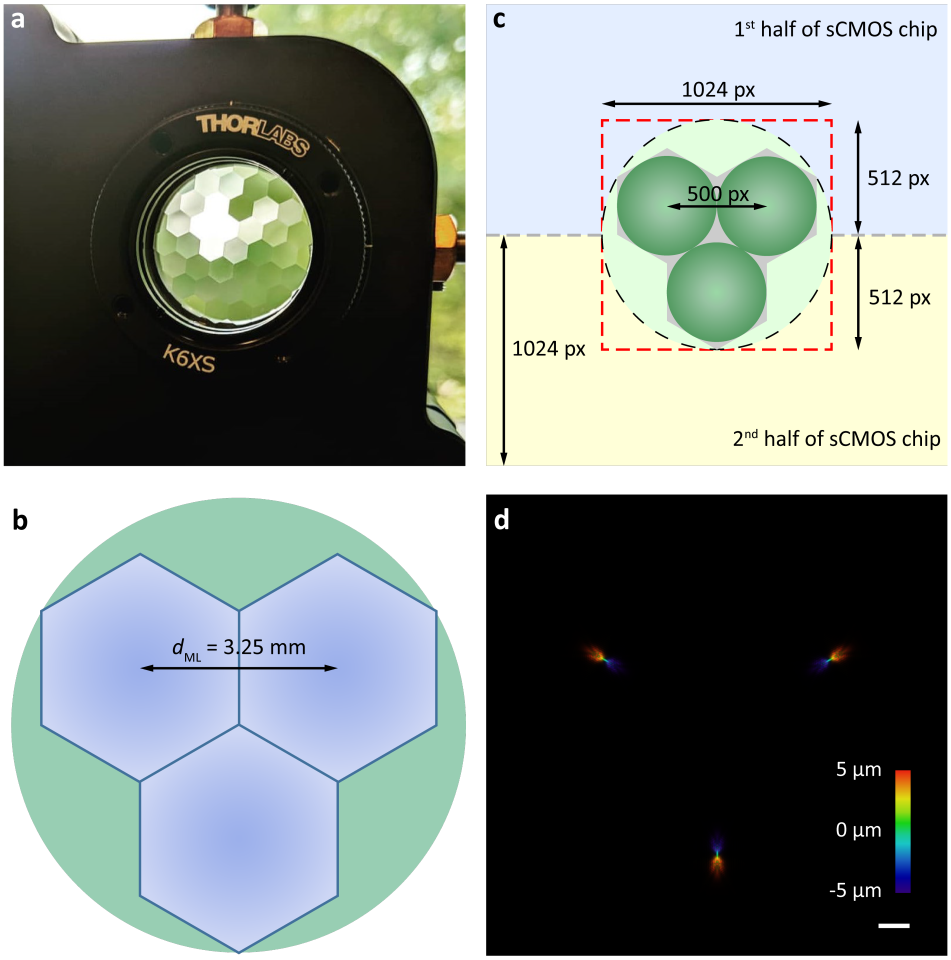

Supplementary Figure 14: Design of the MLA. (a) Photo of the MLA. (b) MLA illustration and light-field partitioning at the Fourier plane. d_{ML} is the distance between the centers of two adjacent microlenses. (c) Elemental images on the camera chip. The three elemental images occupy a 1024-pixel-by-1024-pixel region at the center of the camera sensor. (d) An experimental Fourier LFM PSF in an axial range of -5 µm to 5 µm. Scale bar: 10 µm.

The Fourier light-field imaging part of the LFC is an aperture-partition system, where an MLA is placed to segment the Fourier plane. Such configuration significantly determines the LFM resolution, the field of view (FOV), and the depth of focus (DOF). Theoretically, the MLA was designed to minimize the Fourier aperture segmentation for a sufficient photon budget. By maximizing the FOV and high spatial frequency usage throughout the pupil, the reconstruction quality is secured with an optimum 3D resolution. Hence, we customized the MLA especially for subcellular imaging performance according to

the optical model (Supplementary Note 4). The hexagonal MLA segments the pupil into three off-axis elements with a pitch size of d_{ML}=3.25 mm, an f-number of 36, and a microlens focal length of f_{ML}=117 mm (Supplementary Fig. 14a, b). The light field of a single emitter passing through the MLA occupies the central quarter of the camera sensor (Supplementary Fig. 14c) and forms a PSF with three elemental images (Supplementary Fig. 14d). Here, we omitted the on-axis microlens to reduce segmentation since the on-axis element contains mainly the DC component of the light field with a low angular sensitivity, which contributes less significantly to the overall 3D imaging capability.

Supplementary Note 2. Flow-focusing scheme

As shown in Fig. 1b and Supplementary Fig. 1, we conducted the flow-focusing methods following the hydrodynamic focusing principles[3, 4]. In the experiments, we injected the samples into the main sample channel of the microfluidic chip and red HBSS into the two side channels with a microfluidic pump (OB1 MK4, Elveflow). We adjusted the pressures in the three channels to control the widths of the sample flow and the sheath fluids so that the sample flow width could be constrained within the field-of-view (FOV) of the imaging system. Before the acquisition, we set the pressure to 300~600 mbar for the main sample channel and 600 mbar for each side channel until we could observe a stable hydrodynamic flow where the three channels were merged. The width of the central branch was ~80 μm with these pressure configurations. Once the fluids were stabilized, we decreased the pump pressures proportionally to reduce the cell speed. For imaging with 100 μs exposure time, we set the pressure of the central channel to 12~18 mbar and side channels to 24 mbar. For imaging with 5 μs exposure time, we put the pressure of the central channel to ~400 mbar and side channels to ~600 mbar.

Supplementary Note 3. Setup of stroboscopic illumination

We set up the stroboscopic illumination scheme to shorten the illumination period within a camera exposure period (T_{cam}). Therefore, the effective camera exposure time (T_{eff}) was determined by the illumination period, with which cell movements could be constrained to less than half of the theoretical spatial resolution within T_{eff}. Quantitatively, the displacement d of a sample during T_{eff} can be calculated as $d = v \times T_{eff}$, where v is the flow speed of the sample. To suppress motion blur, it is essential that this displacement d be less than the lateral resolution of our LFC system at 400-600 nm. For experiments with $T_{eff} = 100$ µs, we constrained the sample speed to approximately 3 mm per sec, below the maximum allowable speed of 4-6 mm per sec, derived using $d = v \times T_{eff}$ based on the resolution. Similarly, for experiments with T_{eff} down to 5 µs, the flow speed was set to approximately 115 mm per sec, approaching the maximum allowable speed of 120 mm per sec. As seen, under both conditions, the sample displacement captured within one stroboscopic frame remained shorter than the lateral resolution of LFC, thereby mitigating motion blur. As a result, the motion blur could be effectively suppressed for fast-moving cells (Fig. 1c). In this experiment, we used a 200 Hz frame rate for image acquisition. The rolling shutter mode of the camera allowed for a 600-µs global exposure time for a 1024-pixel-by-1024-pixel frame. For multi-color imaging, the signal frequency of the two-color laser lines was set to be half of the camera frame rate (i.e., 100 Hz), and the two laser wavelengths were switched on and off alternately. Here, we designed two stroboscopic illumination schemes to adapt to various throughput requirements (Supplementary Fig. 15).

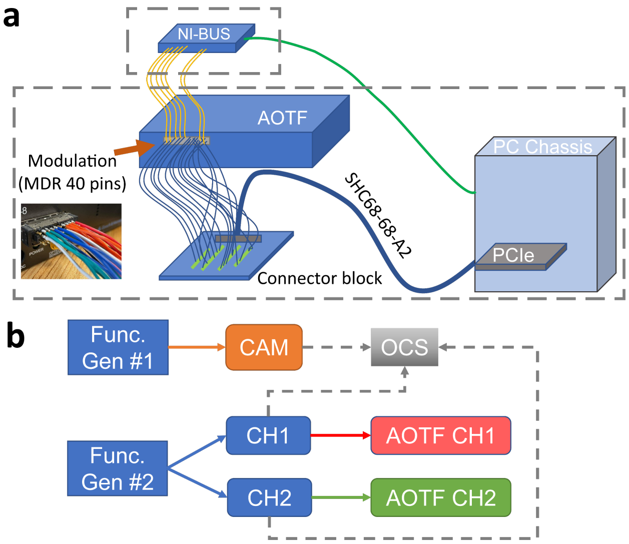

Supplementary Figure 15: Stroboscopic illumination schemes. (a) Schematic using NI DAQ devices. AOTF: acoustic optical tunable filter. NI-BUS: USB 6210 multifunctional I/O device. PCIe: PCIe-6738 Analog Output Device. MDR: mini delta ribbon. (b) Schematic of using function generators and oscilloscopes. Func. Gen: function generators. CAM: sCMOS camera. OCS: oscilloscope. CH1, CH2: Channel 1 and Channel 2 of the function generator. AOTF CH1, AOTF CH2: the Channel 1 and Channel 2 of the acoustic optical tunable filter.

3.1. Using NI DAQ devices

For experiments with a cell speed within 10 mm/s, we used NI DAQ devices to conduct stroboscopic illumination (Supplementary Fig. 15a). An analog signal waveform was generated by a PCIe board (PCIe-6738, National Instruments) and sent by a connector block (CB-68LP, National Instruments) to the acoustic optical tunable filter (AOTF, 97-03926-12, Crystal Technology). At the same time, it was synchronized with the camera signals by a LabVIEW program. Then a BUS (USB-6210, National Instruments) device was used to collect the digital signals from the camera as a feedback source. The DAQ devices above are small and easy to implement into a desktop workstation or an optical system, while they only support an illumination period of no less than 100 μs. For higher-throughput imaging, the signal waveforms generated from DAQ devices become unstable.

3.2. Using function-generating devices

We used two function generators (AFG3102, Tektronix) and an oscilloscope (DPO5104, Tektronix) to perform stroboscopic illumination instead (Supplementary Fig. 15b) for higher-throughput imaging. Rather than using camera signals from its internal trigger, we used the external trigger mode of the camera and drove the camera acquisition with a function generator. Since the internal clocks of the function generators were relatively stable with no significant phase shifts within 3-4 rounds of data acquisition (15~20 min), we set up the two function generators to control the camera and the AOTF, respectively. An oscilloscope was used to monitor the waveforms and the synchronization between two function generators. In the experiments, we set the square wave frequency as 200 Hz, peak width as 4.5 ms for the function generator controlling the camera, and the square wave frequency as 200 Hz, peak width as 5 μs for the other one controlling the AOTF. Therefore, we increased our theoretical throughput by >50-fold with no noticeable motion blur compared with the method described in Supplementary Note 3.1.

Supplementary Note 4. Vectorial Debye model

We derived the wave function at the native image plane (NIP) in Supplementary Equation (1) using the vectorial Debye theory to address the high numerical aperture (NA) of the objective lens and the corresponding refractive-index mismatch (RIM) between the objective lens immersion medium and the sample solution.

$$\mathbf{U_i}(\mathbf{x},\mathbf{p}) = \frac{M}{f_{obj}^2\lambda^2}\int_0^\alpha \sqrt{\cos\theta_1}\sin\theta_1 \times \exp\left[i\mathbf{k_0}\Phi(l) + \frac{i\mathbf{u}\cos\theta_2}{4\sin^2(\alpha/2)}\right]$$

$$\times\left\{(\tau_s + \tau_p\cos\theta_2)J_0\left[\frac{\sin\theta_1}{\sin\alpha}\mathbf{v}\right] - (\tau_s - \tau_p\cos\theta_2)J_2\left[\frac{\sin\theta_1}{\sin\alpha}\mathbf{v}\right]\right\}\mathrm{d}\theta_1 \qquad (1)$$

where f_{obj} is the objective focal length. J_0 and J_2 are the zeroth and second-order Bessel functions of the first kind, respectively. $\mathbf{v}$ and $\mathbf{u}$ represent normalized radial and axial coordinates. The two variables are defined by $\mathbf{v} = \mathbf{k_1}[(x_1/M - p_1)^2 + (x_2/M - p_2)^2]^{1/2}\sin(\alpha)$ and $\mathbf{u} = 4\mathbf{k_2}p_3\sin^2(\alpha/2)$. $\mathbf{p} = (p_1, p_2, p_3) \in \mathbb{R}^3$ is the point source in the object space; $\mathbf{x} = (x_1, x_2) \in \mathbb{R}^2$ represents the image position on the NIP. M is the objective magnification; α is determined by the minimum half-angle of NA and the critical angle of total internal reflection, i.e., $\alpha = min[\sin^{-1}(\mathrm{NA}/n_1), \sin^{-1}(n_2/n_1)]$. The wavenumbers $\mathbf{k_{1,2}} = 2\pi n_{1,2}\hat{\mathbf{k}}_{1,2}/\lambda$ were calculated using emission wavelength λ, refractive index n_1 of the immersion medium and refractive index n_2 of the sample solution. θ_1 and θ_2 are the refractive (objective side) and incident (sample side) angles at the media interface, respectively.

In Supplementary Equation (1), the aberration function $\Phi(l)$, the Fresnel transmission coefficients τ_s and τ_p were defined as $\Phi(l) = -l(n_1\cos\theta_1 - n_2\cos\theta_2)$, $\tau_s = \frac{2\sin\theta_2\cos\theta_1}{\sin(\theta_1+\theta_2)}$ and $\tau_p = \frac{2\sin\theta_2\cos\theta_1}{\sin(\theta_1+\theta_2)\cos(\theta_1-\theta_2)}$, where l is the normal focusing position (NFP). In our imaging system, the refractive index of the immersion oil n_1 is 1.515, and n_2 is 1.33 for the PBS solution. For computational convenience, we set the azimuthal angle of the emitter in the polar coordinates φ_{p} = 90° since the

fluorescence from the emitters exhibited an isotropic polarization so that the light field only pointed to the $\mathbf{p}_1$ direction.

Next, the image at the NIP $\mathbf{U_i}(\mathbf{x},\mathbf{p})$ was optically Fourier transformed onto the back focal plane of the Fourier lens, described as $\mathrm{O}\mathcal{F}\mathrm{T}[\mathbf{U_i}(\mathbf{x},\mathbf{p})]$, which was then modulated by the MLA. The modulation is described by the transmission function $\phi(\mathbf{x}')$, where $\mathbf{x}' = (\mathrm{x}_1', \mathrm{x}_2') \in \mathbb{R}^2$ represents the coordinates on the MLA. Specifically, we describe the microlens aperture as a hexagonal amplitude mask $\mathrm{hex}(\mathbf{x}'/d)$, combined with a phase mask $\exp\left(\frac{-ik}{2f_{\mathrm{ML}}}\|\mathbf{x}'\|_2^2\right)$, where $k = 2\pi/\lambda$ is the wavenumber in the air. The modulation induced by a microlens is then described as:

$$\phi(\mathbf{x}') = \mathrm{hex}(\mathbf{x}'/d)\exp\left(\frac{-ik}{2f_{\mathrm{ML}}}\|\mathbf{x}'\|_2^2\right) \tag{2}$$

Thus, the entire modulation can be described by the convolution of $\phi(\mathbf{x}')$ with a comb function $\mathrm{comb}_{\triangle}(\mathbf{x}'/d)$ that corresponds to the three microlenses (Supplementary Fig. 14), i.e., $\Phi(\mathbf{x}') = \phi(\mathbf{x}')$, where $\otimes$ represents the convolution operator.

The light field propagation from the MLA to the camera can be modeled using the Fresnel propagation over a distance of f_{ML}:

$$\mathbf{h}(\mathbf{x}'',\mathbf{p}) = \mathcal{F}^{-1}\left\{\mathcal{F}[\mathrm{O}\mathcal{F}\mathrm{T}[\mathbf{U_i}(\mathbf{x},\mathbf{p})]\,\Phi(\mathbf{x}')] \times \exp\left[i2\pi f_{ML}\sqrt{\left(\frac{1}{\lambda}\right)^2 - \left(f_x^2 + f_y^2\right)}\right]\right\} \tag{3}$$

where $\mathbf{x}'' = (x_1'', x_2'') \in \mathbb{R}^2$ represents the positions on the camera plane. The exponential term is the Fresnel transfer function. f_x and f_y are the spatial frequencies in the camera plane. $\mathcal{F}$ and $\mathcal{F}^{-1}$ represent the Fourier transform and inverse Fourier transform, respectively. In practice, the Fresnel propagation over distance f_{ML} was calculated in small steps for computational accuracy. The final intensity image $\mathrm{O}(\mathbf{x}'')$ at the camera plane containing elemental images of each microlens is described by:

$$\mathrm{O}(\mathbf{x}'') = \int |\mathbf{h}(\mathbf{x}'',\mathbf{p})|^2\, \mathrm{g}(\mathbf{p})\, dp \tag{4}$$

where $\mathrm{g}(\mathbf{p})$ is the intensity distribution of the volume of isotropic emitters in the object space.

Supplementary Note 5. Reconstruction algorithm using hybrid PSF

As shown in Supplementary Equation (4), the intensity image at the camera image can be described as $O = Hg$, where the measurement matrix H is determined by the PSF. The elements $h_{j,k}$ in H represent the light projection from the k-th volume $g^{(k)}$ in the object space to the pixel $\mathrm{O}(j)$ on the camera plane. Therefore, the reconstruction becomes an inverse problem of retrieving the radiant intensity at each point of the 3D object volume g with the camera image O, which can thus be derived as:

$$g^{(k+1)} = \mathrm{diag}\left[\mathrm{diag}\left(H^T H g^{(k)}\right)^{-1} \left(H^T O\right)\right] g^{(k)} \tag{5}$$

where the operator diag diagonalizes a matrix. This expression is a modified deconvolution algorithm based on the Richardson-Lucy iteration scheme. In our case, the sampling pixel size for reconstruction are Δ_{xy} = 153 nm and Δ_{z} = 100 nm. For visualization, we additionally interpolated 2×2×2 pixels into each reconstructed pixel to match the camera pixel size.

The 3D deconvolution iteratively performs forward projection ($Hg^{(k)}$) and back projection ($H^T O$ and $H^T H g^{(k)}$) between the 3D object space and the 2D camera plane. Here, the spatially invariant 3D PSF $\mathrm{PSF}(\mathbf{x}'', z) = |\mathbf{h}(\mathbf{x}'', \mathbf{p})|^2$ can be described and numerically derived by an on-axis emitter $\mathbf{p} = (0, 0, z)$. As a result, the forward projection can be obtained by summing up the 2D convolutions on each layer within an axial range $[z_0, z_1]$, i.e., $Hg^{(k)} = \sum_{z=z_0}^{z=z_1} \mathrm{PSF}(\mathbf{x}'', z) \otimes \mathrm{g}^{(\mathrm{k})}(z)$, where $\mathrm{g}^{(\mathrm{k})}(z)$ represents a single layer located at *z* in the 3D object volume. Hence, The back projection can be given as $[H^T O](z) = \mathrm{PSF}'(\mathbf{x}'', z) \otimes O$ and $\left[\mathrm{H}^{\mathrm{T}}\mathrm{H}\mathrm{g}^{(\mathrm{k})}\right](z) = \mathrm{PSF}'(\mathbf{x}'', z) \otimes Hg^{(k)}$, where $\mathrm{PSF}'(\mathbf{x}'', z)$ is acquired by rotating $\mathrm{PSF}(\mathbf{x}'', z)$ by 180 degrees.

To be further mentioned, the spatial positions of the elemental images of the numerical PSF were adjusted based on the experimental results to compensate for any instrumental misalignment between the theoretical model and the actual optical system (Supplementary Fig. 16). Moreover, the use of such hybrid PSF values circumvents the intensity value discreteness of the experimental PSF since the sCMOS-recorded images are unsigned integer values representing the photon numbers acquired by the camera chip. On the contrary, the numerical PSF images provide a double precision to estimate the PSF better, thereby enhancing the accuracy of the 3D information retrieval of the object.

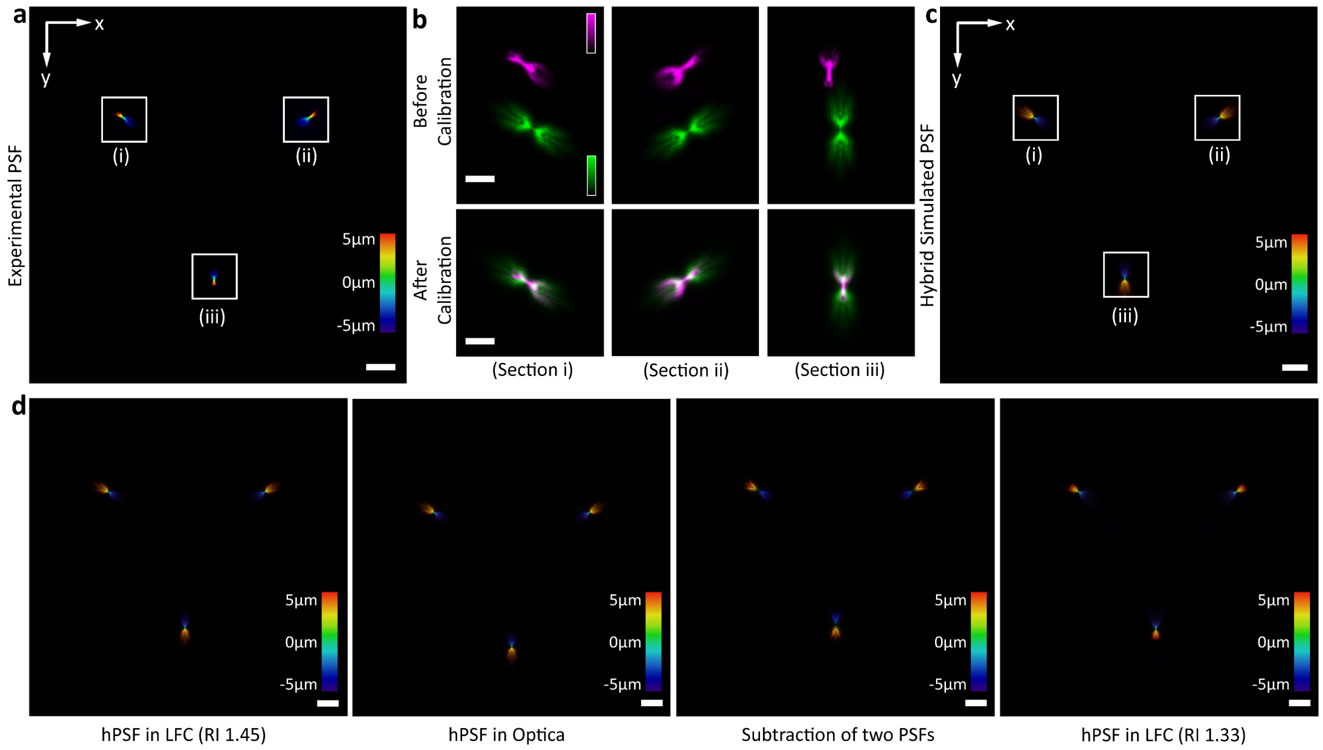

Supplementary Figure 16: PSF calibration. (a) Experimental PSF of Fourier light-field microscopy. (b) PSF calibration between experimental PSF and simulated PSF. Magenta: experimental PSF. Green: simulated PSF. (c) Hybrid PSF (hPSF) after calibration. (d) Comparisons among hPSF in LFC, hPSF in previous work and hPSF in a different refractive index. Scale bars: 10 μm (a, c, d), 5 μm (b).

In practice, we calculated the Fourier transform (i.e., the optical transfer function, OTF) of the PSF as $\mathrm{OTF} = \mathcal{FT}\{\mathrm{PSF}\}$ in advance to enhance the computational efficiency before loading them into the GPU for iterative computation of forward and back projections. Specifically, the forward projection can be performed as $Hg^{(k)} = \sum_{z=z_0}^{z=z_1} \mathcal{F}^{-1}\{\mathrm{OTF}(\mathbf{x}'', z) \cdot \mathrm{G}^{(\mathrm{k})}(z)\}$, where $\mathrm{G}^{(\mathrm{k})}(z)$ represents the Fourier

transform of a single layer at z in the 3D object volume. Similarly, the back projection can be conducted as $[\mathrm{H^T O}](z) = \mathcal{F}^{-1}\{\mathrm{OTF}'(\mathbf{x}'', z) \cdot \mathcal{F}\{O\}\}$ and $[\mathrm{H^T Hg^{(k)}}](z) = \mathcal{F}^{-1}\left\{\mathrm{OTF}'(\mathbf{x}'', z) \cdot \mathcal{F}\{Hg^{(k)}\}\right\}$, where $\mathrm{OTF}'(\mathbf{x}'', z)$ is the transposed $\mathrm{OTF}(\mathbf{x}'', z)$.

To further improve the spatial resolution of our LFC system, we have implemented *rad*FLFM method using the radiality of the light-field images[5]. The method achieves a better spatial resolution beyond the theoretical Fourier light-field resolution without compromising the throughput (Supplementary Fig. 17).

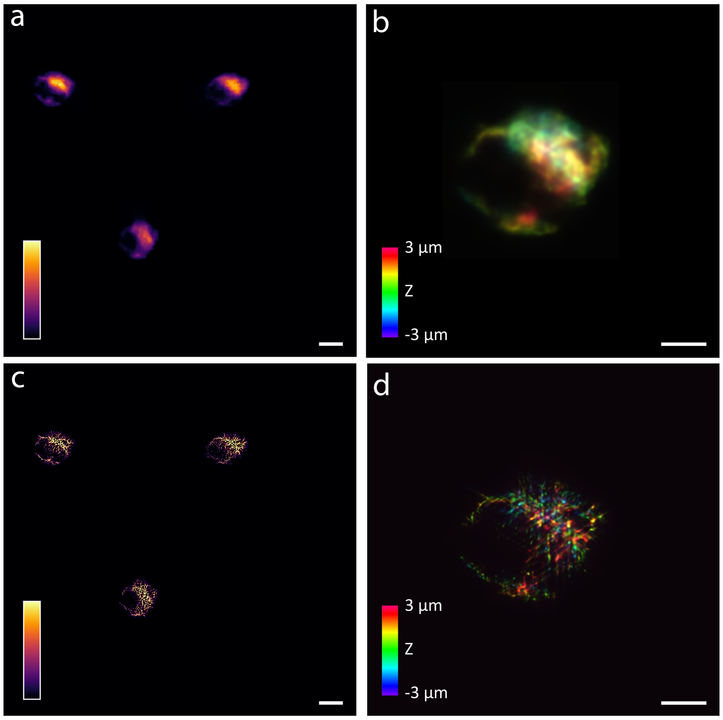

Supplementary Figure 17: Comparisons of reconstructions between with and without radiality analysis. (a) Raw light-field images of mitochondria in flowing HeLa cells. (b) The 3D reconstruction of (a). The depth information was color-coded according to the color scale bar. (c) Corresponding light-field image in (a) using ACsN and radiality analysis. (d) The corresponding 3D reconstruction of (c). Scale bars: 10 μm (a, c), 5 μm (b, d).

Additionally, we have made substantive advancements in our algorithmic framework, specifically incorporating deep neural networks for the task of image reconstruction. To elaborate, traditional Richardson-Lucy deconvolution algorithms have been replaced by deep-learning algorithms optimized for 3D light-field image retrieval. Typically, using the conventional Richardson-Lucy deconvolution (RLD) method requires 30-50 iterations to retrieve the volumetric details of samples with near-

diffraction-limited resolution, which can take around 8-10 seconds. This can be quite time-consuming when dealing with terabytes of IFC data. However, by utilizing a deep learning approach and a well-trained U-Net network, 3D volume prediction can be achieved in as little as 60-80 milliseconds. This means that the deep-learning-powered reconstruction is over 100 times faster than the conventional RLD method, making it possible to quickly analyze large data sets.

For the training dataset, we utilized a collection of 500 previously acquired wide-field volumes featuring HeLa peroxisomes (Supplementary Fig. 18a). These volumes were subjected to deconvolution using a 3D wide-field PSF to enhance their SNR, thereby serving as our ground truth (GT), shown in Supplementary Fig. 18b. Subsequently, these deconvolved wide-field volumes were convolved with a 3D light-field PSF to generate synthetic light-field images. The resultant elemental images were segmented and compiled along the channel dimension to create the training input for the neural network (Supplementary Fig. 18c). The network architecture employed is based on the U-Net framework, as depicted in (Supplementary Fig. 18d). To accommodate the GPU memory constraints of our workstation, the training inputs were resized to dimensions of 512 × 512 × 3 pixels, while the ground truths were resized to 512 × 512 × 64 pixels. The voxel dimensions are set at 130 nm × 130 nm × 65 nm. The network underwent 500 training epochs, completed in an approximate time span of 5-6 hours, utilizing an Nvidia TITAN RTX graphics card for computation. The deep learning-generated reconstructions of (Supplementary Fig. 18e, h, k) are presented in (Supplementary Fig. 18f, i, l) and are compared with corresponding wide-field scanning results in (Supplementary Fig. 18g, j) and Richardson-Lucy deconvolution (RLD) results in (Supplementary Fig. 18m). The quality of the deep learning-reconstructed image is found to be comparable to that achieved through wide-field scanning results and deconvolved results. The intensity values were normalized to a 0-1 scale. The image quality of the deep learning results was measured with 3D structure similarity indices (3D SSIM) and peak signal-to-noise ratios (PSNR).

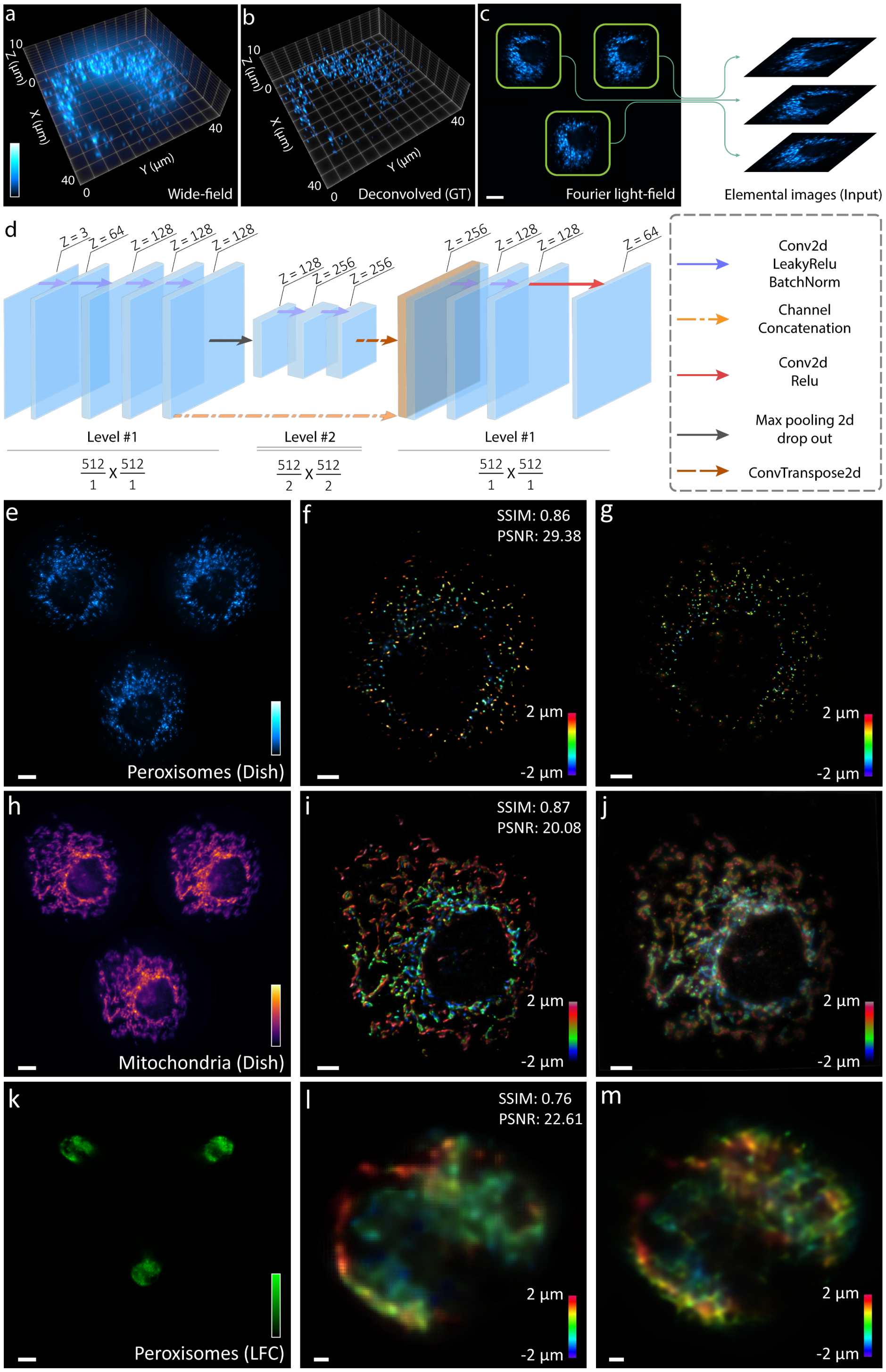

Supplementary Figure 18: Implementation of deep learning scheme. (a) The wide-field z-scanning volume of dish-cultured peroxisomes. (b) The 3D deconvolution volume of (a). (c) The elemental images were segmented and stacked into a channel dimension. (d) The architecture of the network. Z represents the depths of the tensors while the heights and widths of the tensors are marked below the level numbers. (e, h, k) The light-field image of dish-cultured HeLa cell peroxisomes (e), mitochondria (h), and flowing HeLa cell peroxisomes (k). (f, i, l) The deep-learning reconstructed volume of (e, h, k), respectively. (g, j) The wide-field scanning results of the same cell in (e, h). (m) The RLD-reconstructed volume of (k). Scale bars: 10 µm (c, e, h, k), 5 µm (f, g, i, j) ,1 µm (l, m).

Supplementary Note 6. Jurkat cell nucleus imaging with higher throughput

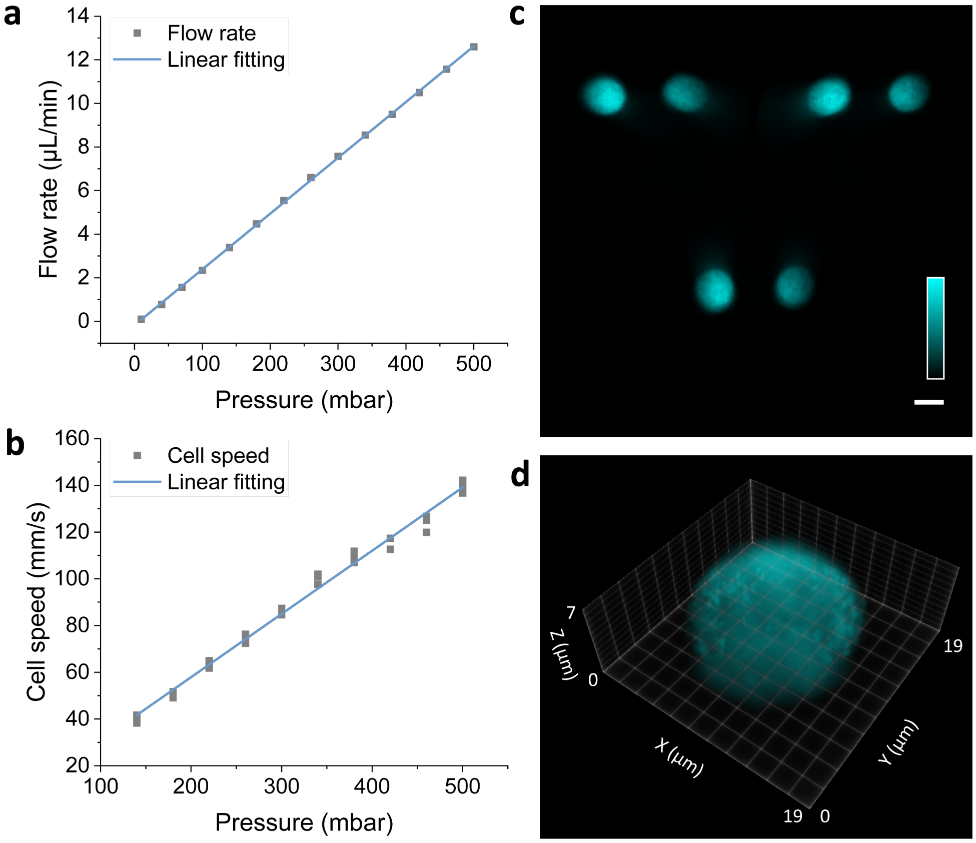

Supplementary Figure 19: Higher throughput imaging with Jurkat cell nucleus. (a) Scatter plot for flow rate measured by microfluidics sensor and pump pressure with linear fitting. (b) Scatter plot for cell speed measured with acquired image sequences and pump pressure with linear fitting. (c) Light-field raw image of a Jurkat cell with the dual-snapshot scheme. (d) Visualization of 3D reconstruction of the Jurkat cell in (c). Scale bar: 10 μm. Source data are provided as a Source Data file.

Using function generators and an oscilloscope, we achieved a more stable stroboscopic illumination in our LFC system, allowing for imaging with a higher throughput. Here, we took advantage of our large field of view (more than twice the size of a Jurkat cell) and implemented a dual snapshot scheme to image a single cell twice in one frame (Supplementary Fig. 19 and Supplementary Movie 15). By controlling the time interval of two snapshots and measuring the distance between two shots, we can accurately estimate the cell speed, which can be further used to estimate the theoretical cell throughput. In practice, we first calibrated the relations between microfluidic pump pressures and flow rates read from a microfluidic flow sensor (MFS, Elveflow). The linear trend of the curve (Supplementary Fig. 19a)

allowed linear interpolation when we set the pressures to specific numbers for a desired flow rate. Secondly, we maintained the distance between two snapshots at ~150 pixels on the camera and calibrated the relations between pump pressures and cell speeds. The linear relations between them (Supplementary Fig. 19b) also allowed interpolation between the calibrated data points.

After the calibrations, we set the microfluidic pump pressure to 414 mbar for the main sample channel and 622 mbar for the side channels to allow a flow rate of ~10.87 μL/min. We set the peak interval of the square wave signals to 239.81 μs and the peak width to 5 μs. The results shown in Supplementary Fig. 19c, d exhibit no noticeable motion blur and can be well reconstructed volumetrically within the field of view and a depth of focus of ~5 μm. The distance between the two snapshots is 190 pixels, equivalent to 27.55 μm. Therefore, the cell speed is estimated to be ~115 mm/s. Assuming the cell-to-cell interval in the flow is around twice the cell size (~20 μm), the theoretical cell throughput we can achieve in our LFC system is ~5,750 cells/s.

To be mentioned, a shorter stroboscopic time reduces motion blur but may weaken the signal-to-noise ratio (SNR), owing to the reduced photon count during the effective exposure time. To address this trade-off, we have implemented our lab-written algorithms, including both background rejection and ACsN[1]. While ACsN has previously been validated for its efficacy in restoring low-SNR light-field images[5-8], its deployment in flow setting has not been demonstrated. In addition, we have also rigorously optimized fluorescent staining protocols, selecting dyes and proteins through multiple rounds of testing to ensure robust results. As a result, our experimental data corroborate that even when utilizing stroboscopic illumination periods as brief as 5-μs, our approach is proficient at reliably restoring raw fluorescent light-field signals for precise 3D image reconstruction (Supplementary Fig. 19). It should be mentioned that efficient illumination and denoising have been primarily employed for recent high-throughput 2D IFC techniques, typically utilizing stroboscopic illumination durations of 10-20 μs[9, 10]. It is worth noting that hardware solutions such as VIFFI[11] or FIRE[12] have also been proposed by high-throughput 2D IFC

techniques to recover a high SNR, however, at the expense of increased instrumental complexity. Furthermore, we have conducted additional experiments with various stroboscopic illumination periods. These experiments were accompanied by a quantitative analysis evaluating the SNR and image quality, both with and without the implementation of ACsN denoising (Supplementary Fig. 20). Our results substantiate that our strategy significantly elevates the quality of reconstructed images. This approach provides compelling evidence supporting the optimized parameters we have employed in the LFC system, effectively balancing robust SNR with minimal motion blur.

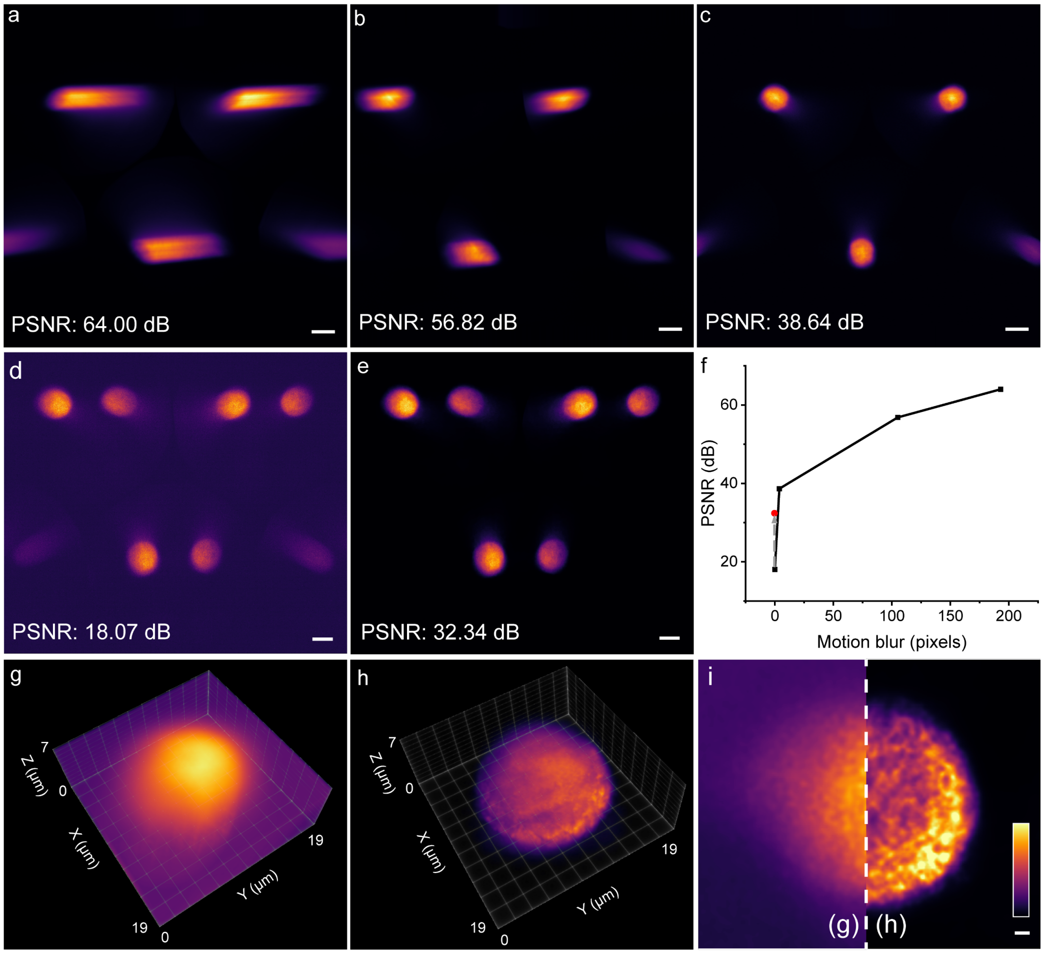

Supplementary Figure 20: Image quality in different noise and motion-blur levels and the effects of using ACsN method. (a-d) Light-field images of the nucleus of flowing Jurkat cells with 5-ms (a), 2.7-ms (b), 100-μs (c), and 5-μs (d) effective exposure time. (e) ACsN denoised light-field image of (d). (f) The relationship between motion blur and SNR. Black lines and dots represent images without ACsN, and the red dot represents the image with ACsN. The gray dashed arrow shows the improvement of SNR by the denoising algorithm. (g, h) The 3D reconstruction of (d) and (e), respectively. (i) 2D images on the focal plane of (g) and (h), respectively. Scale bars: 10 μm (a-e), 1 μm (i). Source data are provided as a Source Data file.

Supplementary Note 7. Imaging system characterization

7.1. Spatial resolution

7.1.1. Lateral resolution

We calculated the lateral resolution of the imaging system as:

$$R_{xy} = \frac{1}{M} \times \frac{\lambda}{2\mathrm{NA}_{\mathrm{ML}}} \times \frac{f_{FL}}{f_{ML}} \tag{6}$$

where M is the objective magnification, and λ is the fluorescence wavelength. $\mathrm{NA}_{\mathrm{ML}}$ is the numerical aperture of each microlens. The focal lengths of the Fourier lens and the microlenses are represented as f_{FL} and f_{ML}, respectively. Our imaging system uses a 100× objective lens with NA = 1.45, λ is 680 nm for dark red fluorescence, 599 nm for orange fluorescence, and 510 nm for green fluorescence. The focal length of the Fourier lens is f_{FL} = 275 mm. The NA of each microlens can be calculated as $\mathrm{NA}_{\mathrm{ML}} = \frac{d_{ML}}{2f_{\mathrm{ML}}} = 0.014$. Therefore, the lateral resolution is 575 nm for dark red fluorescence, 507 nm for orange fluorescence, and 432 nm for green fluorescence, consistent with the measurements of phantom samples (Fig. 2b-e) and biological samples (Fig. 3c, i). Note that the analytical model is derived based on elemental images in the raw Fourier light-field data, while the deconvolution process provides a moderate enhancement in the resolution. When estimating the sizes of the phantom samples, we calculated the phantom volumes $\boldsymbol{V}$ and converted the values to diameters using $D = \sqrt[3]{6V/\pi}$. The distributions reveal that the resolving capability of the LFC system is ~500 nm (Fig. 2f-k).

7.1.2. Axial resolution

We assume that the axial positions of the two emitters can be resolved if they can be resolved laterally in the elemental images. Therefore, we calculated the axial resolution of the imaging system as:

$$R_z = \frac{1}{M^2} \times \frac{\lambda}{2\mathrm{NA}_{\mathrm{ML}}} \times \frac{f_{FL}}{f_{ML}} \times \frac{1}{\tan\theta'} \tag{7}$$

where $\tan\theta' = d_{\mathrm{ML}}/(\sqrt{3}f_{\mathrm{FL}})$. In this case, the axial resolution can be obtained as 843 nm for dark red fluorescence, 743 nm for orange fluorescence, and 632 nm for green fluorescence, consistent with the measurements of phantom samples (Fig. 2b-e) and biological samples (Fig. 3d, j).

Note that it is feasible to apply advanced strategies, such as light-field super-resolution algorithms (e.g., VsLFM[13] or HyLFM[14]) or digital adaptive optics (J. Wu, et al. Cell, 2021[15]) to enhance the resolution during the post-processing process. Specifically, VsLFM enhances resolution by leveraging multiple angles of views scanned by piezo-steering mirrors combined with a digital adaptive optics algorithm. In addition, HyLFM employs an additional light-sheet illumination, facilitating the simultaneous capture of high-resolution images that serve as the training and validation datasets for deep learning networks. We expect both techniques to achieve effectiveness in resolution enhancement. We illustrated these potential strategies as integrated into the LFC system (Supplementary Fig. 21a and b). It should also be noted that it would be the first integration of these methods into Fourier light-field and cytometric imaging fashions.

7.2. Depth of focus (DOF)

Here we treated the full width of the axial PSF (i.e., 2× full width at half maximum (FWHM) in the axial direction) as the DOF of the system, which, therefore, can be calculated as:

$$\mathrm{DOF} = \frac{4R_{xy}^2}{\lambda}\left(2 + \frac{P_{eff}}{R_{xy}}\right) \tag{8}$$

P_{eff} is the effective pixel size of the elemental image, which is 153 nm based on the configuration of our setup. Hence, the DOF is obtained as 4.41 μm for dark red fluorescence, 3.95 μm for orange fluorescence, and 3.44 μm for green fluorescence, which is consistent with the measurements of bead phantoms (Fig. 2b-e) and biological samples (Fig. 3g). Note that the deconvolution in the reconstruction process using PSF considering spherical aberration can retrieve the diffracted information moderately outside of the

Rayleigh range of the axial PSF. Therefore, the LFC presents a depth of field (DOF) of ~6 μm in experimental results (Fig. 3 and 4).

To enhance the coverage, three practical solutions (1-3) can be feasibly executed to extend the DOF in the further development of the LFC system. These solutions include the implementation of (1) a low-magnification objective lens, (2) an additional Fourier light-field path, and (3) an electrically tunable lens (illustrated in Supplementary Fig. 21c-e). In particular, the initial alteration (1) involves transitioning to a 40× objective lens (e.g., Nikon CFI Plan Fluor 40×, 1.3NA Oil). This switch is concomitant with adjustments in the design parameters for both the micro-lens array (MLA) and the Fourier lens[16] (Supplementary Fig. 21c). Here, we propose the parameters for the MLA (f_{ML} = 55.8 mm, pitch d = 3.3 mm, 7 hexagonal microlenses) and the Fourier lens (f_{FL} = 150 mm). Based on our theoretical model, these modifications lead to ~1.5-fold improvement in the DOF (i.e., ~8.2 μm), 3× expanded field of view (220 μm), 3D resolution of 600-850 nm and 1.1-1.5 μm in the lateral and axial dimensions, respectively. In the alternative solution (2), we propose a multi-focal Fourier light-field design to enhance the DOF by placing an additional Fourier light-field path with an offset of 40 mm away from the native image plane so that the two Fourier light-field paths simultaneously capture connective focal ranges (Supplementary Fig. 21d). Last but not least, the solution (3) will replace the normal tube lens with a focus tunable lens (e.g., an electrically tunable lens or ETL) so that different depth layers can be refocused corresponding to the focal changes of the ETL (Supplementary Fig. 21e). By synchronizing the focal scan of the ETL and camera frames, the DOF can be efficiently extended by accumulating the images acquired from multiple frames.

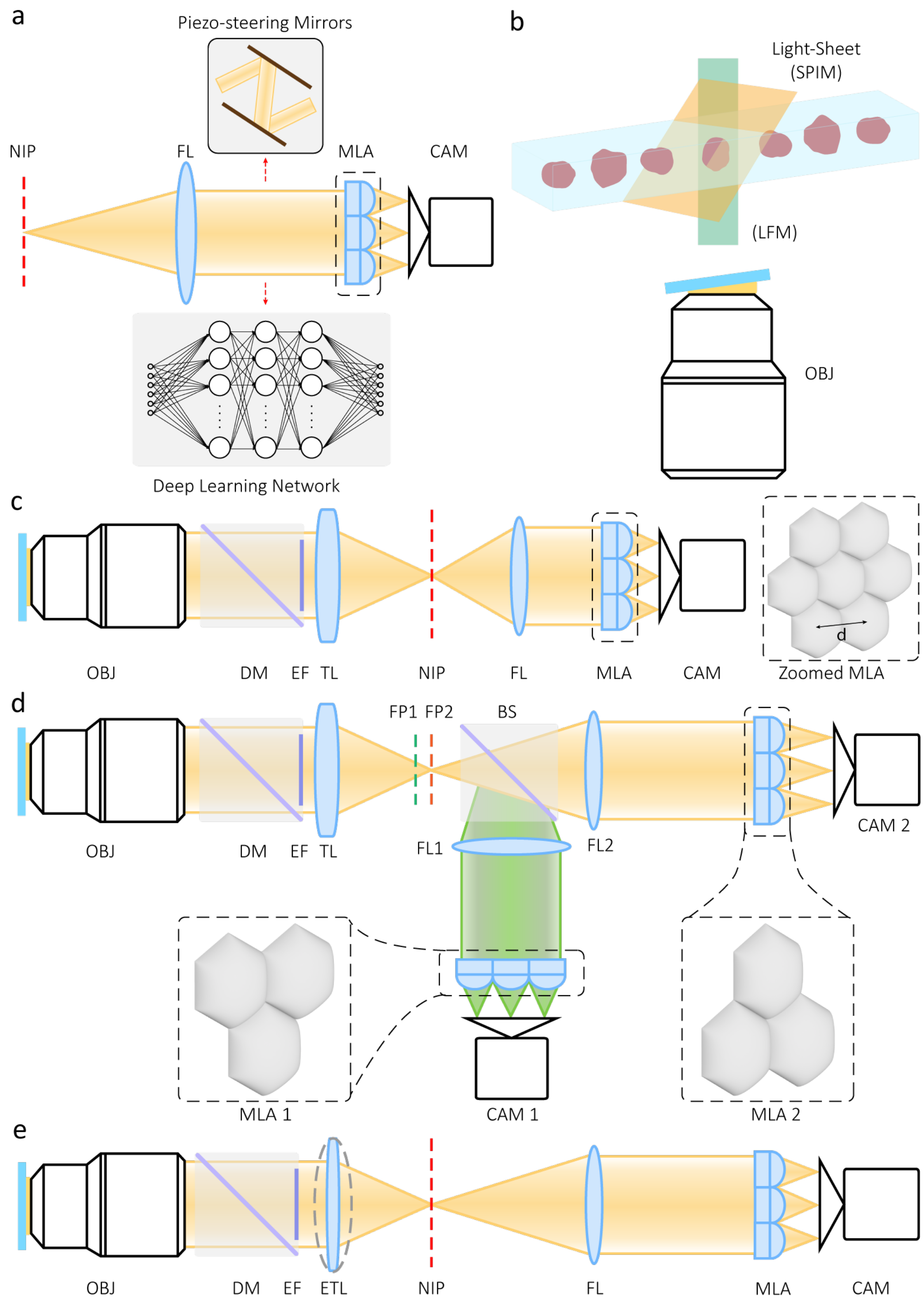

Supplementary Figure 21: Experimental diagrams. (a) Scheme of integrating VsLFM to LFC, which implements piezo-steering mirrors into the Fourier light-field acquisition. The tomographic images taken will be sent to a deep learning network for training, so the scanning images can eventually be virtually predicted. (b) Scheme of integrating HyLFM to LFC, which concomitantly acquires light-sheet image stacks serving as continuous training and validation for deep learning network reconstructing the LFC data. (c-e) The proposed solutions (1-3) to DOF extension. NIP: Native image plane. FL: Fourier lens. MLA: microlens array. SPIM: selective plane illumination

microscopy. LFM: light-field microscopy. OBJ: objective lens. DM: dichroic mirror. EF: emission filter. TL: tube lens. FP: Fourier light-field paths. ETL: electrically tunable lens.

7.3. Field of view (FOV)

The FOV was calculated at the focal plane in the object space of the system:

$$\mathrm{FOV} = d_{ML} \times \frac{f_{FL}}{f_{ML}} \times \frac{1}{M} \tag{9}$$

Using the system configuration parameters, we got a FOV of 76.39 μm × 76.39 μm, which is consistent with the measurements (~70 μm × 70 μm) of our previous results[6]. Supplementary Table 1 summarizes the system parameters and the design principles of MLA.

Supplementary Table 1. System parameters and MLA design principles

Input parameters		Design parameters		
Emission wavelength λ (nm)	516, 599, 680 (Use 600 for calculations)	Effective pupil size at the MLA D_{pupil} (mm)	$2f_{FL} \cdot \frac{\mathrm{NA}}{M}$	7.98
Objective magnification M	100×	Fourier lens focal length f_{FL} (mm)	$\frac{M \cdot D_{pupil}}{2NA}$	275
Numerical aperture NA	1.45	Occupancy ratio N	$\frac{2NA}{\lambda} \cdot R_{xy}$	2.45
Camera pixel size P_{cam} (μm)	6.5	Microlens diameter d_{ML} (mm)	$\frac{D_{pupil}}{N}$	3.25
Physical size of the camera sensor D_{cam} (mm)	13.3	Microlens focal length f_{ML} (mm)	$\frac{S_r \cdot P_{cam} \cdot d_{ML}}{\lambda}$	117

7.4. Temporal resolution

We achieved an effective exposure time of 100 μs without any noticeable motion blur using the NI DAQ devices and 5 μs when using function generators and an oscilloscope. Therefore, we claimed a temporal

resolution of up to 5 μs with our current system configurations. The temporal resolution is mainly constrained by the responding limit of the AOTF.

7.5. Analytical cell throughput

As shown in Supplementary Fig. 22, assuming the cell flow is continuous and stable with a constant cell-to-cell interval, the cell throughput can be directly defined by the following equation:

$$N_c = \frac{v_c}{\Delta_c} = \frac{\delta \cdot \eta}{\Delta_c} \tag{10}$$

where N_c is cell throughput, v_c is cell speed, Δ_c is cell-to-cell interval size, δ is the cell displacement between adjacent frames, and η is camera frame rate.

Our system can achieve a cell speed of up to 115 mm/s. Assuming the cell-to-cell interval is twice the cell size (i.e., approximately 20 μm), we claim an analytical throughput of 5,750 cells/s. In multi-color imaging experiments, the relative distance of two cells between two adjacent frames was measured to estimate the cell speed. For example, in Fig. 3, the relative distance between mitochondria and peroxisome images was measured to be 17.11 μm, equivalent to a flowing speed of 3.4 mm/s.

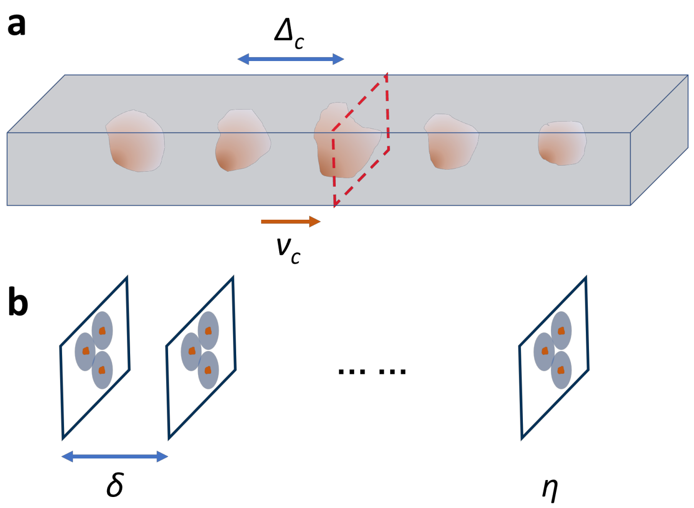

Supplementary Figure 22: cell throughput estimation. (a) Illustration of cells flowing in the microfluidics channel at a speed of v_c. Δ_c represents the cell-to-cell interval. (b) Frame sequence during the high-throughput acquisition.

δ represents the moving distance of cells between two adjacent frames (i.e., within one camera exposure time). η represents the camera frame rate.

7.6. Spatial resolution versus throughput

In essence, high throughput is one primary advantage of IFC over traditional single-cell platforms. This major advantage leads to processing and analyzing thousands to millions of cells in a single experiment, orders of magnitude higher than conventional imaging techniques. Specifically, high throughput offers high content, multiparametric analysis, and statistical significance for large-scale cell studies and screening applications. These single-cell details allow for identifying genes, pathways, and cell biological mechanisms at the population level underlying disease diagnosis in clinical settings[17, 18]. The high number of cells analyzed per sample increases the statistical power and reduces the impact of sample bias in the experiments, which is crucial for detecting subtle and rare phenotypic changes and for robust data interpretation in biological research. The automated nature of cytometric imaging allows for rapid sample loading, data acquisition, and analysis, reducing the time and labor required for experiments. This efficiency is vital in high-throughput screening and large-scale studies. The throughput of IFC also enables cell studies that necessitate imaging of fresh clinical samples or in their native state post-extraction from organs (e.g., Fig. 6). In this case, confocal or super-resolution microscopy becomes limited. The high throughput system can be integrated with other technologies, such as mass spectrometry or genomics platforms, providing a more comprehensive analysis of the cellular state.

On the other hand, LFC overcomes the trade-off between resolution and throughput that is inherent in existing IFC systems. High-resolution imaging provides detailed insights into subcellular structures, yet this is often at the cost of throughput. Conversely, systems tailored for enhanced throughput may compromise resolution. Theoretically, analytical throughput decreases quadratically to the increase in magnification (i.e., resolution) due to constraints imposed by the effective pixel size and the maximal flow

velocity that precludes motion blur (see Fig. 1c)[10]. Conventional approaches have typically achieved high or super-resolution imaging by considerably restricting throughput[19-21]. However, LFC marks a significant advance by formulating an effective magnification of 42.5× (thereby enhancing the throughput) by combining the 100× objective lens with individual microlenses. LFC then restores the near-diffraction-limited resolution, characterized by the 100× objective lens, through the wave-optics-based reconstruction of elemental light-field images. This combinatorial strategy represents a substantial advance in alleviating the resolution-throughput tradeoff for IFC while retaining the unique snapshot 3D ability of light-field imaging, which, as a result, collectively surpasses the analytical throughput of conventional wide-field systems (Supplementary Fig. 23).

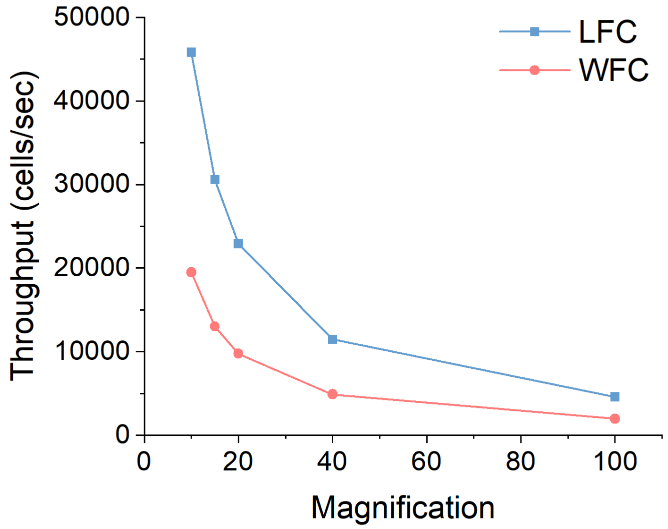

Supplementary Figure 23: Variation in the analytical throughput of LFC and conventional 2D wide-field cytometry (WFC) as a function of objective magnification (10×, 15×, 20×, 40×, 100×). In addition to the 3D ability, LFC achieves a higher throughput owing to the use of the microlens array that results in an effectively lower magnification. The reconstruction of elemental images allows for the recovery of the full resolution. Source data are provided as a Source Data file.

Supplementary Note 8. Comparison with structured illumination microscopy for HeLa cells

We performed additional experiments and analysis by imaging mitochondria and peroxisomes in HeLa cells across multiple platforms. In particular, we compared the results generated by light-field microscopy (Nikon Eclipse Ti2U; OBJ: 100×/1.45NA), wide-field microscopy (the same Nikon Eclipse Ti2U setup as for LFC), and commercial 3D-SIM microscopy (Zeiss LSM 780 with Zeiss ELYRA PS.1; OBJ: 100×/1.46NA, images acquired and processed with Zeiss ZEN 2012 SP5). Also, we used cultured HeLa cells and ensured the quantitative comparison of the same cells across multiple modalities. As seen in Supplementary Fig. 24, the 3D light-field results of subcellular structures displayed a high consistency compared with the scanning wide-field stacks and 3D super-resolution results. Furthermore, we employed the 3D structural similarity index measure (SSIM) to quantitatively verify the accuracy of the results. 3D-SSIMs of mitochondria and peroxisomes exhibited (0.83, 0.95; wide-field) and (0.78, 0.82; SIM) of the light-field images in comparison with the wide-field and SIM results, respectively. In addition to our demonstration, we would also like to clarify that the accuracy and fidelity of light-field microscopy techniques have been demonstrated utilizing various sample types and imaging conditions. Also, to address the flow setting in this study, we used hybrid point-spread functions (hPSFs)[6, 7], considering the spherical aberration caused by the depth in the flow and any actual experimental misalignments and imperfections. In summary, we expect these results and elaborations to clarify the high accuracy of 3D structural retrieval of light-field images consistent with the wide-field and super-resolution SIM images.

Indeed, super-resolution techniques such as SIM can attain a sub-diffraction-limited resolution but remain largely incompatible with the flow setting due to their sequential (rather than snapshot and volumetric) acquisition scheme. As a result, the available super-resolution strategies still rely on conventional platforms to trap and acquire static super-resolution images, unable to preserve the

throughput[19, 22]. As another example, we have also developed a technique termed optofluidic scanning microscopy (OSM)[21] based on image-scanning microscopy (a variant of SIM) to achieve the first super-resolution acquisition of flow cells, but the method was still realized at a significantly reduced throughput (a few cells per second)[21]. In contrast, LFC presents about three orders of magnitude of improvement in the throughput and diffraction-limited 3D resolution, which could be the main desirable and long-seeking feature for researchers using IFC. Regarding the further improvement of the 3D resolution, we have recently reported a computational strategy (Han, 2022)[5] based on the radiality of the light-field images to achieve a resolution doubling comparable to SIM (Supplementary Fig. 17).

Regarding the image resolution difference between Fig. 3 and Supplementary Fig. 24, we want to note that (1) The 3D-SIM experiments (Supplementary Fig. 24) were performed in a culture dish, purposefully designed to allow for cell-to-cell identification and comparison with other wide-field and structured illumination modalities. The cells were attached to the substrate and exhibited flat-distributed subcellular morphology in this condition. In contrast, Hela cells in Fig. 3 were captured in the microfluidic environment, where the flowing cells display their native spherical morphology. Mitochondria and peroxisomes are volumetric and densely packed in this condition, complicating the resolved subcellular structures. (2) The exposure times were different in the two cases. In Fig. 3, the cells were imaged with a short exposure time of 100 μs, and in Supplementary Fig. 24, the images were acquired with a considerably 1000× longer exposure time of 100 ms. This resulted in different signals collected and signal-to-noise ratios (SNRs), thus leading to variations in image quality. (3) The cells were prepared using different staining methods, i.e., GFP/Tracker-staining and immuno-staining, for Fig. 3 and Supplementary Fig. 24, respectively (see Supplementary Note 13), which resulted in relatively higher SNR in Supplementary Fig. 24. (4) The spherical aberrations of flowing cells degrade the overall resolution and image quality in Fig. 3, considering the refractive index mismatch for the high-NA

objective lens. Here, we performed numerical simulations to show the influence of aberration on the LFC results (Supplementary Fig. 25). As seen, the simulated Siemens stars at different axial positions (Supplementary Fig. 25a) were convolved with PSFs taken at the surface of the substrate (approximating the condition in Supplementary Fig. 24) and at 10 μm deep into the solution (approximating the condition in Fig. 3), respectively, to generate synthesized light-field images (left columns in Supplementary Fig. 25b and c). The synthesized light-field images were then deconvolved with the corresponding PSFs to get the reconstructed volumes. The image quality was quantitatively measured with the reconstructed depth layers at the corresponding axial positions (middle and right columns in Supplementary Fig. 25b and c). At each axial position, the intensity profiles along the dashed circles of the same size in both conditions were plotted for comparison. As shown in Supplementary Fig. 25d, the intensity profiles of the sample at the surface shows higher contrast and better resolution compared to the sample 10 μm deep in the solution.

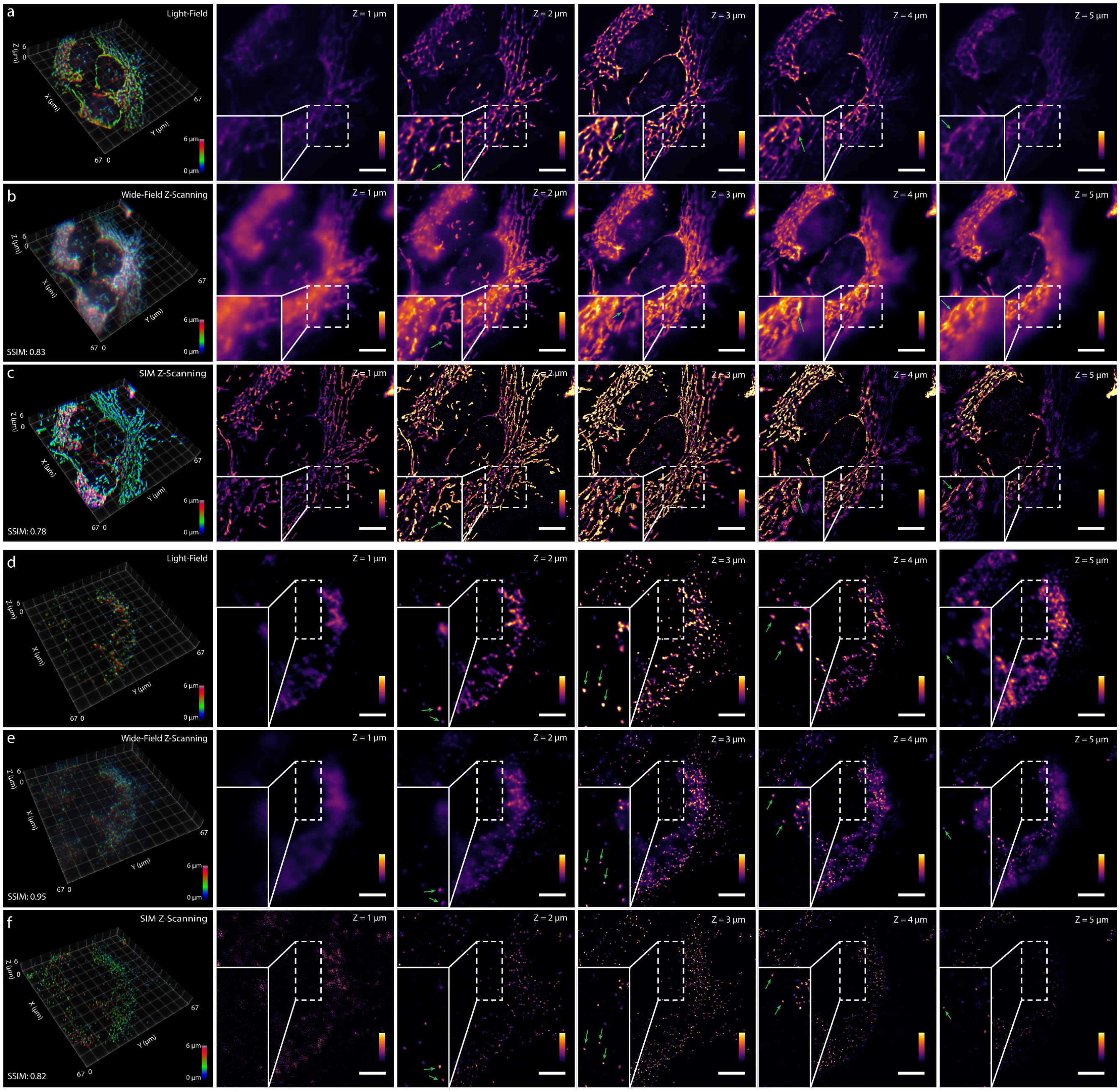

Supplementary Figure 24: Mitochondria and peroxisome imaging using structured illumination microscopy. 3D-rendered (leftmost column) and axial slices at various depths (other columns) of mitochondria (a, b, c) and peroxisome (d, e, f) of HeLa cells, taken by LFC (a, d), scanning wide-field microscopy (b, e), and 3D SIM (c, f). The boxed regions were zoomed in for better visualization and comparison. The green arrows mark the corresponding structures displayed using each imaging method. The 3D structural similarity index measure (SSIM) values were shown in the wide-field and SIM images. Scale bars: 10 µm.

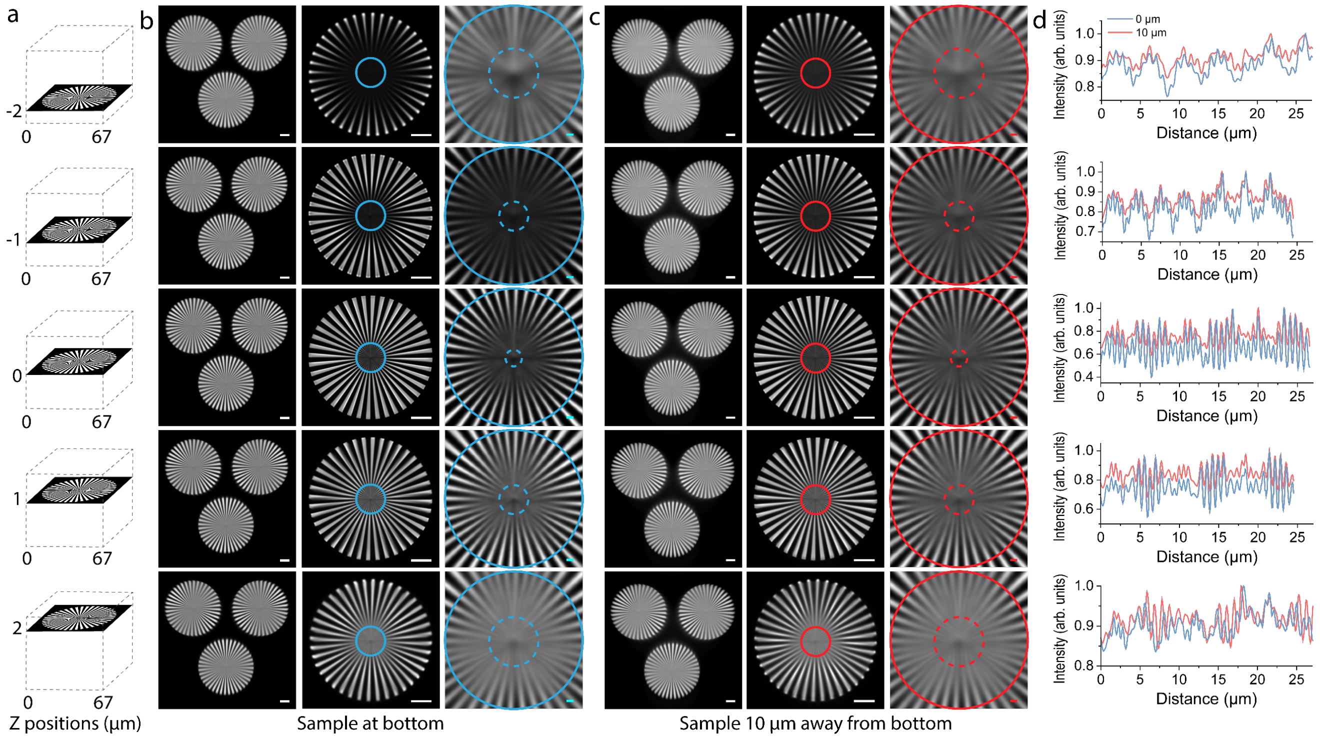

Supplementary Figure 25: Simulation of Siemens stars at different axial locations in the solution. (a) Illustrations of Siemens star patterns at various axial positions away from the focal plane. (b, c) Raw light-field images (left columns), the corresponding z layers of 3D reconstructions (middle columns) with zoomed-in region within the solid-line circles (right columns) of the Siemens stars at the surface of the substrate (b) and 10 μm deep into the solution (c). (d) Intensity profiles along the dashed circles in (b) and (c). Scale bars: 10 μm (b, c, left and middle columns), 1 μm (b, c, right columns). Source data are provided as a Source Data file.

Supplementary Note 9. K-means clustering for blood cell morphology identification

The k-means clustering algorithm is a machine-learning algorithm that aims to partition the observations into k clusters where the elements within the cluster have the smallest distance mean (i.e., nearest mean)[23]. Mathematically, the algorithm is to minimize:

$$\sum_{i=1}^{k}\sum_{x\in X_i}\|x-X_i\|^2 \tag{11}$$

where $X=\{x_1,x_2,\dots,x_i,\dots,x_k\}$ is the clustered dataset.

Using MATLAB functions, we applied this algorithm to identify blood cells with different morphologies. First, we used the radii along three axes from the ellipsoid fitting results as clustering parameters and imperially set the cluster number to be 3. The results identified three types of cells: (A) adhered cell pairs, (B) single spherical cells, and (C) single non-spherical cells. Then we used the eccentricity values along two of the three axes as the clustering parameters to further identify more morphological feathers. We imperially set the cluster number to 2 to further cluster the results from group (A) and group (B). The results showed that for group (A), the algorithm further identified (A1) smaller spherical cells and (A2) larger spherical cells; for group (B), the algorithm further identified (B1) elliptical cells and helmet-shape cells and (B2) stick-like cells and cell fragments. Hence, the entire clustering process identified 5 types of morphologies based on the 3D volumes of the cells.

Supplementary Note 10. Comparison with structured illumination microscopy for Jurkat cells

We performed imaging of staurosporine (STS) treated Jurkat cells across multiple platforms. We utilized light-field microscopy (Nikon Eclipse Ti2U; OBJ: 100×/1.45NA), wide-field microscopy (the same Nikon microscope as for LFC), and commercial 3D-SIM microscopy (Zeiss LSM 780 with Zeiss ELYRA PS.1; OBJ: 100×/1.46NA, images acquired and processed with Zeiss ZEN 2012 SP5). Also, using a lab-derived protocol (Supplementary Note 13) treating the coverslip, we immobilized Jurkat cells to ensure the quantitative comparison of the same cells across multiple modalities. In Supplementary Fig. 26, we measured 3D-SSIMs of mitochondria at 0.87 (0 min after STS treatment), 0.87 (30 min), and 0.89 (120 min) between the wide-field and light-field results. Similar high consistency is also verified between the SIM and light-field results, with 3D-SSIMs of 0.89 (0 min), 0.94 (30 min), and 0.97 (120 min). Consistent measurement (0.80, 0.81, and 0.77 for respective treatment times) was obtained for nucleus imaging using light-field and wide-field microscopy.

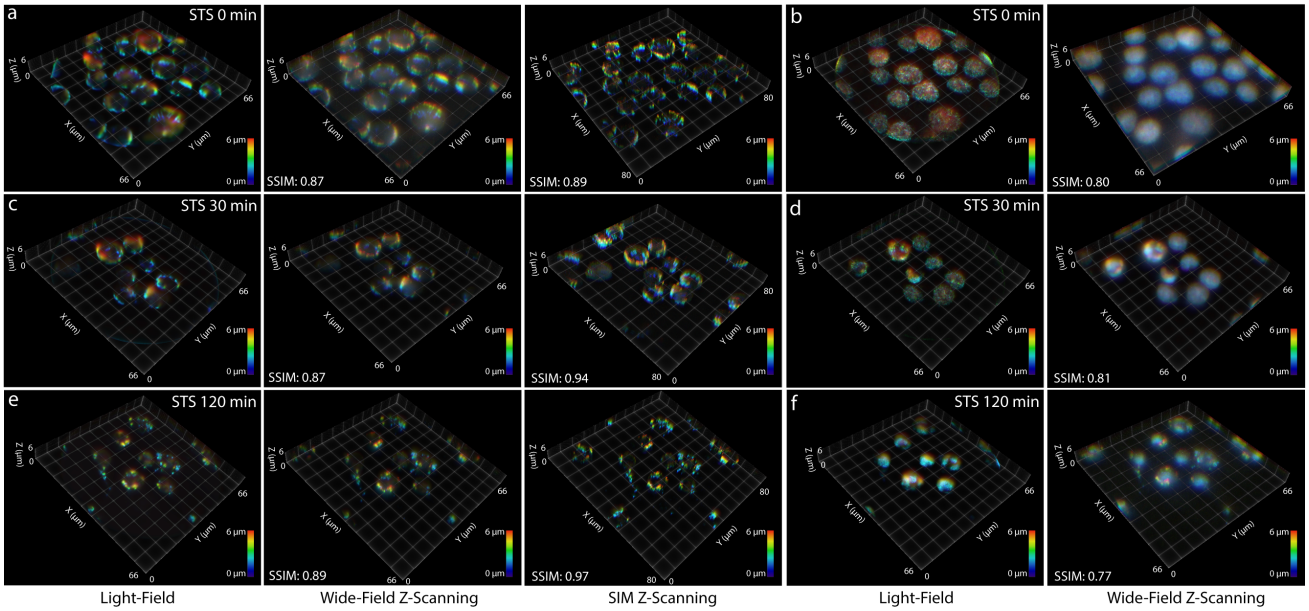

Supplementary Figure 26: Mitochondria and nucleus imaging using structured illumination microscopy. 3D rendering of light-field reconstructions (first and fourth column), wide-field z-scanning stacks (second and fifth column) and SIM z-scanning stacks (third column) of Jurkat cells with staurosporine (STS) treatment. (a, c, e) Jurkat cell mitochondria with 0-min (a), 30-min (c) and 120-min (e) STS treatment. (b, d, f) Jurkat cell nuclei with 0-min (a), 30-min (c) and 120-min (e) STS treatment.

Supplementary Note 11. Comparison with the state-of-the-art IFC techniques

Supplementary Table 2. Comparisons with the state-of-the-art fluorescent IFC techniques

References	3D	Objective (M, NA)	Resolution (nm)	Throughput (objects/sec)	Imaging Instrument
This work – LFC	yes	100×, 1.45	400-600	5,000-10,000	Epi-fluorescence with MLA
Quint, et al., 2017[24]	yes	60×, 1.2	200-300	75-150	Tilted stage, tomography
Fan, et al., 2021[25]	yes	40×, 0.8	1,000	<10	Lattice light-sheet microscopy
Ugawa, et al., 2022[26]	yes	20×, 0.75	880	1200	Strobe light-sheet imaging
Kleiber, et al., 2020[27]	yes	20×, 0.42	700-900	350	3D focusing tomography
Kumar, et al., 2022[28] (VFC/iLIFE)	yes	20×, 0.4	1,786	10-20	Light-sheet microscopy
Han, et al., 2019[29]	yes	10×, 0.28	2,000	500	Scanning light-sheet illumination
Zhang, et al., 2022[30]	yes	10×, 0.28	10,000	500-1,000	Scanning light-sheet illumination
Miura, et al., 2018[31]	yes	20×, 0.75	1,000	10,000	Light-sheet microscopy
Nitta, et al., 2018[32] (IACS)	no	60×, 1.4	2,000	100	FDM confocal microscopy
Holzner, et al., 2021[33]	no	40×, 0.75<br>20×, 0.5<br>10×, 0.5	500-1,000	5,350 (40×)<br>10,900 (20×)<br>20,500 (15×)<br>61,000 (10×)	Light-sheet illumination
Mikami, et al., 2020[11] (VIFFI)	no	20×, 0.75	700	10,000	Polygon scanner light-sheet illumination
Isozaki, et al., 2020[34] (iIACS2.0)	no	20×, 0.75	700	2,000	Polygon scanner
Munoz, et al., 2018[35]	no	20×, 0.45	700-900	7,000-23,000	Beat-frequency multiplexing
ThermoFisher Scientific (Attune CytPix)	no	20×, 0.45	800	6,000	Commercial system
Rane, et al., 2017[9]	no	10×, 0.5 20×, 0.45	700-800	50,000-100,000	Multichannel chip
Gong, et al., 2023[36]	no	20×, 0.4	390	10,000	Anti-diffraction light sheet illumination
Ota, et al., 2018[37] (FiCS)	no	20×	1,000	10,000	Static random light structure Structured illumination/detection
Schraivogel, et al., 2022[38] (ICS)	no	10×, 0.3	1,550	15,000	Radiofrequency–tagged emission (FIRE)
George, et al., 2004[39] (Amnis ImageStream)	no	--, 0.75	500-1,000	1,200 (60×)<br>2,000 (40×)	Commercial system
Goda, et al., 2012[40] (STEAM)	no	--, 0.65	1,400	100,000	Serial time-encoded amplified microscopy

We have generated a coordinates system of the spatial resolution, the throughput, and their 3D imaging capability, based on Supplementary Table 2 (Supplementary Fig. 27).

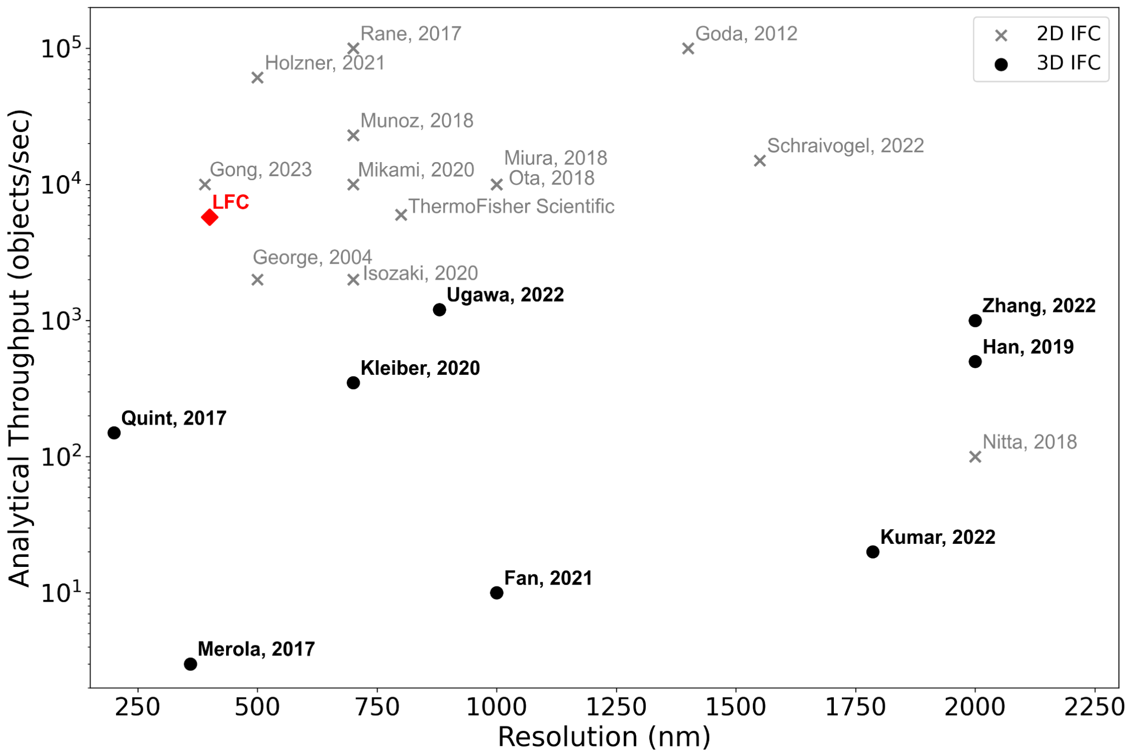

Supplementary Figure 27: State-of-the-art IFC techniques. Red diamond: LFC; Round black dots, 3D IFC techniques. Gray crosses, 2D IFC techniques. Miura, 2018 showed 2D results and claimed the system is capable of 3D imaging.

It is also noteworthy that the light-field cytometer is implemented seamlessly with standard epi-fluorescence microscopy and microfluidic systems. This quality facilitates a platform of ready and high accessibility and relevance for broad existing IFC studies. For example, in this work, we have demonstrated various applications such as particle sorting, cell morphology screening, cell heterogeneity, chemical-induced cell apoptosis, and lipid nanoparticle-enclosed mRNA delivery. The resolution (400-600 nm) has been validated by visualizing subcellular structures such as nuclei, mitochondria, peroxisomes, cytoplasm, and membranes. These demonstrations will contrast existing 3D IFC applications of similar subcellular entities, typically relying on a spatial resolution of 800-1800 nm[25, 28,

[31]. Furthermore, LFC will enable the visualization of many other subcellular features that require IFC, such as spot counting for nanoparticle uptake in cells, calcium location detection in T cells, the activation of eosinophils, blood cell classification, and micronucleus phenotypes identification in cells exposed to a genotoxic compound[41].

Supplementary Note 12. System configurations for image processing

We used a desktop workstation (Intel Xeon W-2145 3.70 GHz Processor, 64.0 GB RAM, Nvidia GeForce GTX 1080 GPU, 2560 NVIDIA CUDA Cores, 8 GB GPU memory, and Samsung Sm961 Series 1 TB Solid State Drive) to perform image acquisition. The data processing and analyses were conducted with another desktop workstation with an Intel Xeon E5-2620 v4 2.10 × GHz Processor, 128 GB RAM, NVIDIA TITAN RTX GPU, 4608 NVIDIA CUDA cores, and 24 GB GPU memory. The numerical PSFs were generated using Python 3.5+ and CUDA C++ (with CUDA 10.1+). We used MATLAB R2021+ with a CUDA-enabled GPU computing kit to perform hybrid PSF implementation and 3D reconstruction. We fully utilized the matrix operations in MATLAB, which helped significantly reduce the use of loops. GPU computing allows the computation to be conducted parallelly. We reconstructed volumes with 101 transverse layers, and the computing time for each transverse plane within a single iteration is less than 2 ms. As a result, we can complete a single iteration of all the layers in less than 0.2 sec. For a 50-iteration 3D reconstruction, the total time consumed (excluding file reading and writing to disk) is about 8 seconds. The microfluidics system was controlled by Elveflow ESI software, and the imaging data was recorded by HCImage. The NI-DAQ-based stroboscopic illumination was controlled by a self-written LabVIEW program. The simulated PSF was generated by a self-written Python program integrated with CUDA C++ and OpenCL. We used MATLAB R2022b, Python 3.9+ (including PyVista[42] for 3D rendering), Origin 2023, and Fiji ImageJ (including the ClearVolume[43] for 3D rendering and 3D ImageJ suite[44] for 3D segmentation) for further data processing and visualization.

Supplementary Note 13. Additional sample preparation

13.1. Mouse platelet acquisition and staining

C57BL/6 mice (N = 2 mice/group) were anesthetized with ketamine and xylazine (doses of 150 mg/kg and 15 mg/kg, respectively). 1 mL of blood was drawn from the vena cava, and the animal was sacrificed upon completion of the procedure. Murine blood was withdrawn into a 1.0 mL syringe containing 100 μL of Clexane (final concentration 40 U/mL). Blood was transferred to an Eppendorf tube and topped with an appropriate amount of 7X acid citrate dextrose (ACD, Sodium Citrate ($2H_2O$) 2.5 g /100 mL 85 mM, Citric Acid (Anhydrous) 1.4 g/100 mL 72.9 mM, D-Glucose 1.5 g/100 mL 110 mM, Theophylline* 70mM) buffer for a final concentration of 1X. After inversion, the blood was placed in a 37°C oven, and blood and all buffers were kept at 37 °C for the remainder of the process. The blood was transferred to a PRP separation tube and centrifuged at 250× g for 2.5 minutes. The platelet-rich plasma (PRP) was removed, and enough platelet washing buffer (PWB, K_2HPO_4 43 mM, Na_2HPO_4 43 mM, NaH_2PO_4 243 mM, NaCl 1.13 M, D-Glucose 55 mM, Theophylline* 100 mM) was added with 20 U/mL Clexane, 0.01 U/mL apyrase, and 5 mg/mL bovine serum albumin (BSA) for a final volume of 1 mL. The PRP was centrifuged at 2000g for 1 minute, the supernatant was discarded, and the pellet was resuspended with PWB containing 20 U/mL Clexane for 1 mL total volume. The platelets were centrifuged again in 1950× g for 1 minute, the supernatant discarded, and the pellet was resuspended with Tyrode's buffer ($NaHCO_3$ 120 mM, HEPES 100 mM, NaCl 1.37 M. KCl 270 mM, D-Glucose 550 mM) containing 0.02 U/mL apyrase. The platelets then rested at 37 °C for 30 minutes before use.

On the day of imaging, 15 μL WGA was added to the platelet vial for 25 min incubation at 37 ℃. Then the solution was washed once with 1.5 mL PBS in the centrifuge with 600× g for 8 min. 1.5 mL 4% PFA was added to perform fixation at room temperature for 12 min. After the fixation, the solution was washed again with 1.5 mL PBS in the centrifuge with 600 × g for 8 min. 3 mL PBS with 5 mM EDTA was added

to the solution before imaging.

13.2. Immunostaining for HeLa peroxisomes and mitochondria

Fixed peroxisome and mitochondria imaging was performed with HeLa cells. The cells were cultured in Dulbecco's modified Eagle medium with 10% fetal bovine serum and 1% PenStrep at 37 °C and in a 5% CO_2 condition. Once reached ~80% confluency, The cells were passaged and cultured in an 8-well glass-bottom μ-Slide with 200 μL of growth medium in each well.

One day prior to the day of imaging, each well was first washed with 500 μL PBS once. Then, each well was fix with 200 μL 3.7% PFA in PBS at 37°C for 12 minutes. Extra aldehyde groups were reduced with 200 μL of 0.1% sodium borohydride, followed by 3 times PBS washing, 5 minutes each. After that, the cells were permeabilized with 0.1% Triton X-100 in PBS for 10 minutes and blocked with 4% bovine serum albumin (BSA) in PBS for 45 minutes. The cells were labeled with TOMM20 Polyclonal Antibody at 2 μg/mL in 0.1% BSA, incubated at 4°C overnight.

On the next day, the cells were then labeled with PMP70 Polyclonal Antibody at 10 μg/mL in 0.1% BSA for 1 hour at room temperature, with light avoided, by simply changing the medium. Next, each well was washed 5 times with 200 μL washing buffer (0.2% BSA with 0.05% Triton X-100 in PBS) for 10 minutes per wash at room temperature. After washing, 150 μL of anti-rabbit and anti-mouse secondary antibody dilutions (final concentration 2 μg/mL; final concentration 2 μg/mL) in blocking buffer (3% BSA with 0.2% Triton X-100 in PBS) was added to each well and incubated for 30 minutes at room temperature, light avoided. Then, each well was washed 3 times with 200 μL washing buffer for 10 minutes per wash at room temperature, followed by one time washing in 500 μL PBS for 5 minutes. For better fluorescence imaging, the cells were post-fixed 200 μL 3.7% PFA in PBS at room temperature for 10 minutes, followed by 3 times washing in 500 μL PBS for 5 minutes per wash. Finally, cells were stored in 500 μL PBS for imaging purposes.

13.3. Floating Jurkat cell mounting onto the imaging dish

Since Jurkat T cells are floating cells, stacking z-depth scanning requires the cells to be mounted firmly. A protocol introduced previously was generally followed and modified to our needs[45]. Briefly, an 8-well glass-bottom µ-Slide was first incubated with 0.1% poly-L-lysine in each well for 10 minutes. Then, the poly-L-lysine was washed off with 70% ethanol, and the coverslips were left to dry. Then, CD3 antibodies (Hit-3a, 10 µg/mL) were added to each well and incubated for 2 hours at 37 °C. Excess anti-CD3 was removed by washing with L-15 imaging media immediately before cell plating. Jurkat T cells were centrifuged (250 g) and resuspended in L-15 medium and then plated in each well with 200 µL of cell solution (~1×10^5 cells). The cells were allowed to settle for 10 minutes. The STS treatment is the same as mentioned in Methods. After treatment, the cells were rinsed with 500 µL PBS three times at room temperature, covered with 100% methanol, cooled to −20 °C for 3 minutes, and, finally, washed three times with 500 µL PBS.

Supplementary Table 3. List of experimental samples and materials

Name	Brand	Category ID
200 nm fluorescent beads	Thermo Fisher Scientific	T7280
1 µm fluorescent beads	Thermo Fisher Scientific	T7282
4 µm fluorescent beads	Thermo Fisher Scientific	T7283
2 µm fluorescent beads	Bangs Laboratories, Inc.	FSFR005, FCSY007, FSDG005
Citrate buffer	Teknova	
cKK-E12	Oragnix Inc.	O-8744
C18PEG2K	Avanti Lipids	
18:1 (Δ9-Cis) PE (DOPE)	Avanti Lipids	
Microfluidic device for LNP formulation	Hamilton Company	
Dynamic light scattering	Wyatt	DynaPro Plate Reader II
20 kD dialysis cassette	Thermo Scientific	
NanoDrop	Thermo Scientific	
1X TE	Thermo Fisher Scientific	
Triton X-100	Fisher BioReagents	BP151-100
RiboGreen reagent	Thermo Fisher Scientific	
Plate reader	BioTek	Synergy H4 Hybrid
Ai14 mice	Jackson Laboratories	C57BL/6J (B6/000664)
Wheat germ agglutinin (WGA)	Thermo Fisher Scientific	W11261
phosphate-buffered saline (PBS)	Corning	21-040-CM
4% paraformaldehyde (PFA) solution	Thermo Fisher Scientific	FB002
16% paraformaldehyde (PFA)	Electron Microscopy Sciences	15710
EDTA	Thermo Fisher Scientific	15575038
trypsin-EDTA	Thermo Fisher Scientific	25200056
HeLa cells	Sigma-Aldrich	93021013
Dulbecco's modified Eagle medium (DMEM)	Corning	10-013-CV
10% fetal bovine serum (FBS)	Corning	35-011-CV
1% Penicillin-Streptomycin (PenStrep)	Thermo Fisher Scientific	15140122
CellLight Peroxisome-GFP	Thermo Fisher Scientific	C10604
MitoTracker Deep Red FM	Thermo Fisher Scientific	M22426
Hank's balanced salt solution (HBSS)	Corning	21-021-CV
Hank's balanced salt solution without Phenol Red	Corning	21-023-CV
CD8+ T cell isolation kit	Stemcell Technologies	
Rosewell Park Memorial Institute medium (RPMI)	Thermo Fisher Scientific	11875101
FluoroDish	World Precision Instruments	FD35-100
SYTO16	Thermo Fisher Scientific	S7578
1.2X HCS CellMask Deep Red	Thermo Fisher Scientific	H32721
Dimethyl sulfoxide (DMSO)	Thermo Fisher Scientific	D12345

Density gradient centrifugation	Lymphoprep	07801
SepMate-15mL tube	STEMCELL technologies	85415
EasySep Human CD3 Positive Selection Kit II	STEMCELL technologies	17851
Dynabeads Human T-Activator	Thermo Fisher Scientific	11131D
X-vivo 10 Serum-free Hematopoietic Cell Medium	Lonza	04-380Q
5% Human AB serum	Valley Biomedical	HP1022
10 mM N-Acetyl-L-cysteine	Sigma Aldrich	A9165
55 µM 2-Mercaptoethanol	Sigma Aldrich	M3148-100ml
50 µ/mL recombinant human IL-2	TECIN™ Teceleukin, NCI	Bulk Ro 23-6019
Collagenase Type I	Sigma Aldrich	
Collagenase XI	Sigma Aldrich	
Hyaluronidase	Sigma Aldrich	
anti-CD31	BioLegend	390
anti-CD45	BioLegend	30-F11
TruStain FcX™ (anti-mouse CD16/32) Antibody	BioLegend	93
Jurkat T cells	Sigma Aldrich	88042803
Staurosporine (STS), 99+%	Thermo Scientific	J62837. #0
SYTO deep red	Thermo Fisher Scientific	S34900
8-well glass-bottom µ-Slide	ibidi USA	80827
sodium borohydride	Sigma-Aldrich	452882
bovine serum albumin (BSA)	Sigma-Aldrich	A7906
TOMM20 Polyclonal Antibody	Thermo Fisher Scientific	PA5-52843
PMP70 Polyclonal Antibody	Thermo Fisher Scientific	MA5-31368
anti-rabbit secondary antibody	Thermo Fisher Scientific	A32723
anti-mouse secondary antibody	Thermo Fisher Scientific	A32733
CD3 antibody (Hit-3a)	Thermo Fisher Scientific	16-0039-81
L-15 imaging media	Thermo Fisher Scientific	21-083-027

Supplementary Table 4. List of LFC experimental configurations

Imaging experiment targets		Beads	HeLa cells	Blood cells	Spleen cells	Mouse T cells
Acquisition configurations	Microscope magnification	100×				
	Excitation wavelength (nm)	488,561,647	488 (peroxisome) 647 (mitochondria)	488	488	488 (nucleus) 647 (membrane)
	Emission wavelength (nm)	512,599,680	512 (peroxisome 680 (mitochondria)	512	512	512 (nucleus), 680 (membrane)
	sCMOS FOV	1024 pixels x 1024 pixels (72.5 μm x 72.5 μm) for HeLa cell imaging with continuous exposure<br>1024 pixels x 900 pixels (72.5 μm x 70.1 μm) for other experiments				
	sCMOS exposure time	5 ms				
	sCMOS frame rate	200 Hz				
	Effective exposure time (μs)	100	5000 (continuous) 100 (stroboscopic)	100	100	100
	Flow rate (μL/min)	0.4-0.6	0.03 (continuous) 0.4-0.6 (stroboscopic)	0.4-0.6	0.4-0.6	0.4-0.6
	Cell speed (mm/s)	3	0.11 (continuous) 3 (stroboscopic)	3	3	3
Processing configuration	ACsN (Yes/No)	Yes				
	Deconvolution iterations	30	80 (continuous) 50 (stroboscopic)	50	50	30 (nucleus) 50 (membrane)
Imaging experiment targets		Human T cells/ HeLa cells	Liver/lung endothelial and spleen cells	Platelets	Jurkat cells	Jurkat cells (fast)
Acquisition configurations	Microscope magnification	100×				
	Excitation wavelength (nm)	488 (nucleus), 647 (membrane)	561 (tdTomato), 647 (membrane)	488	488 (nucleus) 647 (mitochondria)	488
	Emission wavelength (nm)	512 (nucleus), 680 (membrane)	599 (tdTomato), 680 (membrane)	512	512 (nucleus) 680 (mitochondria)	512
	sCMOS FOV	1024 pixels x 900 pixels (72.5 μm x 70.1 μm)				
	sCMOS exposure time	5 ms				
	sCMOS frame rate	200 Hz				
	Stroboscopic exposure time (μs)	100	100	100	100	5
	Flow rate (μL/min)	0.4-0.6	0.4-0.6	0.4-0.6	0.4-0.6	10.87
	Cell speed (mm/s)	3	3	3	3	114.88
Processing configuration	ACsN (Yes/No)	Yes				
	Deconvolution iterations	10 (nucleus), 30 (membrane)	50	30	50	30

Supplementary References

1. Mandracchia, B. et al. Fast and accurate sCMOS noise correction for fluorescence microscopy. *Nature Communications* **11**, 94-94 (2020).
2. Sternberg, S. Biomedical Image Processing. *IEEE Computer* **16**, 22-34 (1983).
3. Lee, G.-B., Chang, C.-C., Huang, S.-B. & Yang, R.-J. The hydrodynamic focusing effect inside rectangular microchannels. *Journal of Micromechanics and Microengineering* **16**, 1024 (2006).
4. Golden, J.P., Justin, G.A., Nasir, M. & Ligler, F.S. Hydrodynamic focusing—a versatile tool. *Analytical and Bioanalytical Chemistry* **402**, 325-335 (2012).
5. Han, K. et al. 3D super-resolution live-cell imaging with radial symmetry and Fourier light-field microscopy. *Biomed. Opt. Express* **13**, 5574-5584 (2022).
6. Hua, X., Liu, W. & Jia, S. High-resolution Fourier light-field microscopy for volumetric multi-color live-cell imaging. *Optica* **8**, 614-620 (2021).
7. Liu, W., Kim, G.R., Takayama, S. & Jia, S. Fourier light-field imaging of human organoids with a hybrid point-spread function. *Biosens Bioelectron* **208**, 114201 (2022).
8. Ling, Z., Han, K., Liu, W., Hua, X. & Jia, S. Volumetric live-cell autofluorescence imaging using Fourier light-field microscopy. *Biomedical Optics Express* **14**, 4237 (2023).
9. Rane, A.S., Rutkauskaite, J., deMello, A. & Stavrakis, S. High-throughput multi-parametric imaging flow cytometry. *Chem* **3**, 588-602 (2017).
10. Holzner, G. et al. High-throughput multiparametric imaging flow cytometry: toward diffraction-limited sub-cellular detection and monitoring of sub-cellular processes. *Cell Rep* **34**, 108824 (2021).
11. Mikami, H. et al. Virtual-freezing fluorescence imaging flow cytometry. *Nat Commun* **11**, 1162 (2020).
12. Diebold, E.D., Buckley, B.W., Gossett, D.R. & Jalali, B. Digitally synthesized beat frequency multiplexing for sub-millisecond fluorescence microscopy. *Nature Photonics* **7**, 806-810 (2013).
13. Lu, Z. et al. Virtual-scanning light-field microscopy for robust snapshot high-resolution volumetric imaging. *Nature Methods* **20**, 735-746 (2023).
14. Wagner, N. et al. Deep learning-enhanced light-field imaging with continuous validation. *Nature Methods* **18**, 557-563 (2021).
15. Wu, J. et al. Iterative tomography with digital adaptive optics permits hour-long intravital observation of 3D subcellular dynamics at millisecond scale. *Cell* **184**, 3318-3332 e3317 (2021).
16. Guo, C., Liu, W., Hua, X., Li, H. & Jia, S. Fourier light-field microscopy. *Optics Express* **27**, 25573-25594 (2019).
17. Gérard, A. et al. High-throughput single-cell activity-based screening and sequencing of antibodies using droplet microfluidics. *Nature Biotechnology* **38**, 715-721 (2020).
18. Boutros, M., Heigwer, F. & Laufer, C. Microscopy-Based High-Content Screening. *Cell* **163**, 1314-1325 (2015).
19. AbuZineh, K. et al. Microfluidics-based super-resolution microscopy enables nanoscopic characterization of blood stem cell rolling. *Sci Adv* **4**, eaat5304 (2018).
20. Almada, P. et al. Automating multimodal microscopy with NanoJ-Fluidics. *Nat Commun* **10**, 1223 (2019).
21. Mandracchia, B., Son, J. & Jia, S. Super-resolution optofluidic scanning microscopy. *Lab on a Chip* (2021).
22. Almada, P. et al. Automating multimodal microscopy with NanoJ-Fluidics. *Nature Communications* **10**, 1223-1223 (2019).
23. Arthur, D. & Vassilvitskii, S. in Proceedings of the eighteenth annual ACM-SIAM symposium on Discrete algorithms 1027-1035 (2007).

24. Quint, S. et al. 3D tomography of cells in micro-channels. *Applied Physics Letters* **111**, 103701 (2017).
25. Fan, Y.-J. et al. Microfluidic channel integrated with a lattice lightsheet microscopic system for continuous cell imaging. *Lab on a Chip* **21**, 344-354 (2021).
26. Ugawa, M. & Ota, S. High-speed 3D imaging flow cytometry with optofluidic spatial transformation. *Biomed. Opt. Express* **13**, 3647-3656 (2022).
27. Kleiber, A. et al. 3-Step flow focusing enables multidirectional imaging of bioparticles for imaging flow cytometry. *Lab on a chip* **20**, 1676-1686 (2020).
28. Kumar, P., Joshi, P., Basumatary, J. & Mondal, P.P. Light sheet based volume flow cytometry (VFC) for rapid volume reconstruction and parameter estimation on the go. *Scientific reports* **12**, 1-15 (2022).
29. Han, Y. et al. Cameraless high-throughput three-dimensional imaging flow cytometry. *Optica* **6**, 1297-1304 (2019).
30. Zhang, Z. et al. A high-throughput technique to map cell images to cell positions using a 3D imaging flow cytometer. *Proc Natl Acad Sci U S A* **119**, e2118068119 (2022).
31. Miura, T. et al. On-chip light-sheet fluorescence imaging flow cytometry at a high flow speed of 1 m/s. *Biomedical optics express* **9**, 3424-3433 (2018).
32. Nitta, N. et al. Intelligent Image-Activated Cell Sorting. *Cell* **175**, 266-276 e213 (2018).
33. Holzner, G. et al. High-throughput multiparametric imaging flow cytometry: toward diffraction-limited sub-cellular detection and monitoring of sub-cellular processes. *Cell Reports* (2021).
34. Isozaki, A. et al. Intelligent image-activated cell sorting 2.0. *Lab Chip* **20**, 2263-2273 (2020).
35. Munoz, H.E. et al. Single-Cell Analysis of Morphological and Metabolic Heterogeneity in Euglena gracilis by Fluorescence-Imaging Flow Cytometry. *Anal Chem* **90**, 11280-11289 (2018).
36. Gong, Y. et al. Flow Cytometry with Anti-Diffraction Light Sheet (ADLS) by Spatial Light Modulation. *Micromachines (Basel)* **14** (2023).
37. Ota, S. et al. Ghost cytometry. *Science* **360**, 1246-1251 (2018).
38. Schraivogel, D. et al. High-speed fluorescence image-enabled cell sorting. *Science* **375**, 315-320 (2022).
39. George, T.C. et al. Distinguishing modes of cell death using the ImageStream® multispectral imaging flow cytometer. *Cytometry Part A: the journal of the International Society for Analytical Cytology* **59**, 237-245 (2004).
40. Goda, K. et al. High-throughput single-microparticle imaging flow analyzer. *Proc Natl Acad Sci U S A* **109**, 11630-11635 (2012).
41. Rees, P., Summers, H.D., Filby, A., Carpenter, A.E. & Doan, M. Imaging flow cytometry. *Nature Reviews Methods Primers* **2**, 86 (2022).
42. Sullivan, C. & Kaszynski, A. PyVista: 3D plotting and mesh analysis through a streamlined interface for the Visualization Toolkit (VTK). *Journal of Open Source Software* **4**, 1450 (2019).
43. Royer, L.A. et al. ClearVolume: open-source live 3D visualization for light-sheet microscopy. *Nature Methods* **12**, 480-481 (2015).
44. Ollion, J., Cochennec, J., Loll, F., Escudé, C. & Boudier, T. TANGO: a generic tool for high-throughput 3D image analysis for studying nuclear organization. *Bioinformatics* **29**, 1840-1841 (2013).
45. Li, X. et al. Three-dimensional structured illumination microscopy with enhanced axial resolution. *Nat Biotechnol* **41**, 1307-1319 (2023).
