# Supplementary material for: Light-field flow cytometry for high-resolution, volumetric and multiparametric 3D single-cell analysis
Source: Nat Commun. 2024 Mar 4;15:1975. doi: 10.1038/s41467-024-46250-7 (PMC10912605; doi:10.1038/s41467-024-46250-7)
Supplement: Supplementary file 3 — Reporting Summary [file 41467_2024_46250_MOESM3_ESM.pdf]

nature portfolio

Corresponding author(s): SHU JIA

Last updated by author(s): Jan 10, 2024

Reporting Summary

Nature Portfolio wishes to improve the reproducibility of the work that we publish. This form provides structure for consistency and transparency in reporting. For further information on Nature Portfolio policies, see our Editorial Policies and the Editorial Policy Checklist.

Statistics

For all statistical analyses, confirm that the following items are present in the figure legend, table legend, main text, or Methods section.

n/a	Confirmed	
☐	☒	The exact sample size (n) for each experimental group/condition, given as a discrete number and unit of measurement
☒	☐	A statement on whether measurements were taken from distinct samples or whether the same sample was measured repeatedly
☒	☐	The statistical test(s) used AND whether they are one- or two-sided<br>*Only common tests should be described solely by name; describe more complex techniques in the Methods section.*
☒	☐	A description of all covariates tested
☒	☐	A description of any assumptions or corrections, such as tests of normality and adjustment for multiple comparisons
☐	☒	A full description of the statistical parameters including central tendency (e.g. means) or other basic estimates (e.g. regression coefficient) AND variation (e.g. standard deviation) or associated estimates of uncertainty (e.g. confidence intervals)
☒	☐	For null hypothesis testing, the test statistic (e.g. F, t, r) with confidence intervals, effect sizes, degrees of freedom and P value noted<br>*Give P values as exact values whenever suitable.*
☒	☐	For Bayesian analysis, information on the choice of priors and Markov chain Monte Carlo settings
☒	☐	For hierarchical and complex designs, identification of the appropriate level for tests and full reporting of outcomes
☒	☐	Estimates of effect sizes (e.g. Cohen's d, Pearson's r), indicating how they were calculated

Our web collection on statistics for biologists contains articles on many of the points above.

Software and code

Policy information about availability of computer code

Data collection	Custom codes with LabVIEW 2021 for PSF acquisition, Hamamatsu HCImage Live 4.5.0.0 for all LFC acquisitions. Zeiss ZEN 2012 SP5 for SIM acquisitions.
Data analysis	Fiji ImageJ with clearvolume-1.4.2 for 3D rendering and 3D ImageJ Suite 4.0.93 for 3D segmentation. Matlab R2022b, Python 3.9 with numpy 1.23.5, tifffile 2022.8.12, imageio2.27.0, dcimg 0.6.0.postl, pyopencl 2021.2.9, pycuda 2021.1.1, cupy 10.5 and pyvista 0.38.4. CUDA C++ and OpenCL codes were generated for PSF simulation. Zeiss ZEN 2012 SP5 for SIM reconstruction. Origin Pro 2023 was used for plotting. Our data analysis codes are available in the provided .zip file and https://github.com/ShuJiaLab/LFC.

For manuscripts utilizing custom algorithms or software that are central to the research but not yet described in published literature, software must be made available to editors and reviewers. We strongly encourage code deposition in a community repository (e.g. GitHub). See the Nature Portfolio guidelines for submitting code & software for further information.

Data

Policy information about availability of data

All manuscripts must include a data availability statement. This statement should provide the following information, where applicable:

- Accession codes, unique identifiers, or web links for publicly available datasets
- A description of any restrictions on data availability
- For clinical datasets or third party data, please ensure that the statement adheres to our policy

The data underlying figures in both main text and supplementary material are available at https://doi.org/10.5281/zenodo.10471580. Additional datasets

supporting this study is available upon reasonable request to the corresponding authors due to a large file size.

Research involving human participants, their data, or biological material

Policy information about studies with human participants or human data. See also policy information about sex, gender (identity/presentation), and sexual orientation and race, ethnicity and racism.

Reporting on sex and gender	Gender was not considered because they are irrelevant. We only used human immune cells (peripheral blood mononuclear cells) for fluorescence staining and imaging, not to any biological aspects of the cells or the donor. Immune cells from any donor work the same regardless of their gender.
Reporting on race, ethnicity, or other socially relevant groupings	Race, ethnicity, or other socially relevant groups are not considered in this study.
Population characteristics	Healthy male and female (not pregnant) adult donors. Human immune cells were used in this study only as a tool to present fluorescence. The property or biology of human immune cells is not the focus of this study.
Recruitment	Participants were recruited on a volunteer basis with full disclosure of the usage of the cells. There was no self-selection bias.
Ethics oversight	The protocols were approved as IRB #H20228 by Georgia Institute of Technology and Emory University Institutional Review Boards.

Note that full information on the approval of the study protocol must also be provided in the manuscript.

Field-specific reporting

Please select the one below that is the best fit for your research. If you are not sure, read the appropriate sections before making your selection.

[x] Life sciences [ ] Behavioural & social sciences [ ] Ecological, evolutionary & environmental sciences

For a reference copy of the document with all sections, see nature.com/documents/nr-reporting-summary-flat.pdf

Life sciences study design

All studies must disclose on these points even when the disclosure is negative.

Sample size	No calculation was used to predetermine the sample size. However, expected sample sizes were estimated to demonstrate that the LFC system can take high-resolution 3D cell images at a high throughput that a conventional 3D imaging flow cytometer cannot achieve. Hence, the sample sizes for analysis were empirically determined to be ≥30 for both reliable measurements of statistics and feasible replications of experiments.
Data exclusions	Due to the large depth of the microfluidics chips and the cell density in the sample solution, images in the data sequences with no objects, highly-defocused objects and cell debris were excluded from the image processing pipelines. For cell imaging, due to the fluorescence intensity variance, images with a maximum value no more than 150 (camera background noise is ~100) were excluded for more accurate statistical analysis. All the exclusion criteria were determined after reviewing images of objects to confirm that the excluded data points were obvious errors.
Replication	The fluorescence staining protocols were repeated at least twice for each experiment. During the data acquisition, samples were loaded and imaged by at least three independent imaging sessions for each experiment.
Randomization	For Jurkat cell apoptosis experiments, the Jurkat cells are evenly mixed and passaged to five groups. Then each group was randomly assigned to a STS treatment period. For other experiments, randomization is not applicable, because no controlled trials were performed.
Blinding	For beads imaging, the investigators blindly mixed the 4 sizes of beads during the acquisition. During data analysis, the investigators preallocated equinumerous beads to difference size groups by visual inspections so that each histogram peak can be adequately plotted. The following volume calculations were performed with all these beads pooled together, and the analysis of all these beads shared the same settings. Thus, the preallocation is irrelevant to the analysis. For Jurkat cell apoptosis experiments, the investigators were blind to apoptotic status of the cells within each STS treatment group. Blinding is not applicable for other data acquisition and analysis process in this study, because no controlled trials were performed.

Reporting for specific materials, systems and methods

We require information from authors about some types of materials, experimental systems and methods used in many studies. Here, indicate whether each material, system or method listed is relevant to your study. If you are not sure if a list item applies to your research, read the appropriate section before selecting a response.

Materials & experimental systems

n/a	Involved in the study
☐	☒ Antibodies
☐	☒ Eukaryotic cell lines
☒	☐ Palaeontology and archaeology
☐	☒ Animals and other organisms
☒	☐ Clinical data
☒	☐ Dual use research of concern
☒	☐ Plants

Methods

n/a	Involved in the study
☒	☐ ChIP-seq
☐	☒ Flow cytometry
☒	☐ MRI-based neuroimaging

Antibodies

Antibodies used	Anti-CD31 (#390, BioLegend, 1:200 dilution), anti-CD45 (#30-F11, BioLegend, 1:200 dilution), TruStain FcX™ Antibodies (anti-mouse CD16/32, #93, BioLegend, 1:100 dilution), CD3 (Hit-3a, #16-0039-81, Thermo Fisher Scientific, 1:100 dilution), TOMM20 (#PA5-52843, Thermo Fisher Scientific, 1:50 dilution), PMP70 (#MA5-31368, Thermo Fisher Scientific, 1:100 dilution).
Validation	Companies that sells the antibodies we used provide quality certificate that certifies these antibodies have been manufactured and tested in accordance with their specifications. The catalog numbers of the antibodies are provided so that each antibodies can be found on the website of each company.<br>Quality Control:<br>BioLegend: https://www.biolegend.com/en-gb/quality<br>Thermo Fisher Scientific: https://www.thermofisher.com/us/en/home/life-science/antibodies/invitrogen-antibody-validation

Eukaryotic cell lines

Policy information about cell lines and Sex and Gender in Research

Cell line source(s)	Hela cells (93021013, Sigma Aldrich)<br>Jurkat T cells (88042803, Sigma Aldrich)
Authentication	According to the suppliers, the cell lines undergo comprehensive quality control and authentication procedures by ECACC, including:<br>•Tested for mycoplasma by culture isolation, Hoechst DNA staining and PCR<br>•Tested for bacteria, yeast and fungi<br>•Species verification by DNA bar-coding and identity verification by DNA profiling<br>•Classical DNA fingerprinting using multi-locus probes is carried out for non-human cell lines
Mycoplasma contamination	According to the suppliers, manufactured cell banks are routinely tested for mycoplasma by ECACC, however no test results were provided. After receiving the cell lines, mycoplasma was not tested.
Commonly misidentified lines (See ICLAC register)	No commonly misidentified cell lines were used in the study.

Animals and other research organisms

Policy information about studies involving animals; ARRIVE guidelines recommended for reporting animal research, and Sex and Gender in Research

Laboratory animals	Ai14 (age of 6-18 weeks), OT-I (age of 6-8 weeks), and C57BL/6 (age of 6-8 weeks) mice were bred at the animal facility in Georgia Institute of Technology. C57BL/6J (age of 8-12 weeks) mice were purchased from Jackson Laboratories.
Wild animals	No wild animals were used in this study.
Reporting on sex	Sex is not necessary to be considered in this study.
Field-collected samples	No field-collected samples were used in this study.
Ethics oversight	All animal experiments were performed following protocols approved by the Institute Animal Care and Use Committee of Georgia Instituted of Technology. All animals were housed in the animal facility at the Institute of Technology.

Note that full information on the approval of the study protocol must also be provided in the manuscript.

Plants

Seed stocks	*Report on the source of all seed stocks or other plant material used. If applicable, state the seed stock centre and catalogue number. If plant specimens were collected from the field, describe the collection location, date and sampling procedures.*
Novel plant genotypes	*Describe the methods by which all novel plant genotypes were produced. This includes those generated by transgenic approaches, gene editing, chemical/radiation-based mutagenesis and hybridization. For transgenic lines, describe the transformation method, the number of independent lines analyzed and the generation upon which experiments were performed. For gene-edited lines, describe the editor used, the endogenous sequence targeted for editing, the targeting guide RNA sequence (if applicable) and how the editor was applied.*
Authentication	*Describe any authentication procedures for each seed stock used or novel genotype generated. Describe any experiments used to assess the effect of a mutation and, where applicable, how potential secondary effects (e.g. second site T-DNA insertions, mosiacism, off-target gene editing) were examined.*

Flow Cytometry

Plots

Confirm that:

☐ The axis labels state the marker and fluorochrome used (e.g. CD4-FITC).

☐ The axis scales are clearly visible. Include numbers along axes only for bottom left plot of group (a 'group' is an analysis of identical markers).

☐ All plots are contour plots with outliers or pseudocolor plots.

☒ A numerical value for number of cells or percentage (with statistics) is provided.

Methodology

Sample preparation	20α-OH cholesterol lipid nanoparticle was formulated using a microfluidic device as previously described. Cre mRNA was diluted in 10 mM citrate buffer. cKK-E12 was purchased from Oragnix Inc. (O-8744). C18PEG2K and 18:1 (Δ9-Cis) PE (DOPE) were diluted in 100% ethanol and purchased from Avanti Lipids. Citrate and ethanol phases were combined in a microfluidic device by syringes at a flow rate of 3:1. See Methods and Supplementary Note 13 for other sample preparation procedures.
Instrument	Elveflow OB1 MK3+
Software	ESI 3.04.01 (Elveflow)
Cell population abundance	Greater than 1000.
Gating strategy	Gating strategy is based on fluorescence staining. See Methods and Supplementary Note 13 for details.

☐ Tick this box to confirm that a figure exemplifying the gating strategy is provided in the Supplementary Information.
