# Supplementary material for: Light-field flow cytometry for high-resolution, volumetric and multiparametric 3D single-cell analysis
Source: Nat Commun. 2024 Mar 4;15:1975. doi: 10.1038/s41467-024-46250-7 (PMC10912605; doi:10.1038/s41467-024-46250-7)
Supplement: Supplementary file 4 — Description of Additional Supplementary Files [file 41467_2024_46250_MOESM4_ESM.pdf]

Description of Additional Supplementary Files

File Name: Supplementary Software 1

Description: The supplementary software package contains all the codes used for reconstructing and analyzing LFC images. The package is structured into 6 key steps shown as follows:

Step 1 contains codes for the conversion between .dcimg files and .tiff files and the reading and writing functions of the 16-bit TIFF file.
Step 2 contains codes for selecting LFC frames with cells.
Step 3 contains codes for light-field point spread function correction.
Step 4 contains codes for the initial stages of data handling (denoising, image cropping,
and padding), 3D reconstruction, and color-coded stack visualization.
Step 5 contains codes for further data analysis and is structured by figures.
Step 6 contains codes for volume rendering methods for 3D stacks using MATLAB, Python, and Fiji ImageJ.

File Name: Supplementary Movie 1

Description: Wide-field and light-field images of flowing fluorescent beads (200 nm, 1 µm, 2 µm, and 4 µm) with multiple laser lines. The magenta color represents 680-nm fluorescence. The green color represents 599-nm fluorescence. The cyan color represents 516-nm fluorescence. Scale bar: 10 µm.

File Name: Supplementary Movie 2

Description: Two-color wide-field images of peroxisomes and mitochondria in flowing HeLa cells. The magenta color represents mitochondria with 680-nm fluorescence. The cyan color represents peroxisomes with 516-nm fluorescence. Scale bar:10 µm.

File Name: Supplementary Movie 3

Description: Two-color light-field images of peroxisomes and mitochondria in flowing HeLa cells. The magenta color represents mitochondria with 680-nm fluorescence. The cyan color represents peroxisomes with 516-nm fluorescence. Scale bar:10 µm.

File Name: Supplementary Movie 4

Description: Wide-field images of membrane-labeled mouse blood cells.
Scale bar: 10 µm.

File Name: Supplementary Movie 5

Description: Light-field images of membrane-labeled mouse blood cells.
Scale bar: 10 µm.

File Name: Supplementary Movie 6

Description: Wide-field images of membrane-labeled mouse spleen cells.
Scale bar: 10 µm.

File Name: Supplementary Movie 7

Description: Light-field images of membrane-labeled mouse spleen cells.
Scale bar: 10 µm.

File Name: Supplementary Movie 8

Description: Two-color wide-field images of mitochondria and nucleus of Jurkat cells under different treatment conditions. The magenta color represents mitochondria with 680-nm fluorescence. The cyan color represents the nucleus with 516-nm fluorescence. Scale bar: 10 µm.

File Name: Supplementary Movie 9

Description: Two-color light-field reconstructed volumes of mitochondria and nucleus of Jurkat cells with no treatment. The magenta color represents mitochondria with 680-nm fluorescence. The cyan color represents the nucleus with 516-nm fluorescence.

File Name: Supplementary Movie 10

Description: Two-color light-field reconstructed volumes for mitochondria and nucleus of Jurkat cells with 30-min STS treatment. The magenta color represents mitochondria with 680-nm fluorescence. The cyan color represents the nucleus with 516-nm fluorescence.

File Name: Supplementary Movie 11

Description: Two-color light-field reconstructed volumes for mitochondria and nucleus of Jurkat cells with 60-min STS treatment. The magenta color represents mitochondria with 680-nm fluorescence. The cyan color represents the nucleus with 516-nm fluorescence.

Supplementary Movie 12
Description: Two-color lightfield reconstructed volumes for mitochondria and nucleus of Jurkat cells with 120-min STS treatment. The magenta color represents mitochondria with 680-nm fluorescence. The cyan color represents the nucleus with 516-nm fluorescence.

Supplementary Movie 13
Description: Two color lightfield reconstructed volumes for mitochondria and nucleus of Jurkat cells with 300-min STS treatment. The magenta color represents mitochondria with 680-nm fluorescence. The cyan color represents the nucleus with 516-nm fluorescence.

Supplementary Movie 14
Description: Two-color lightfield images of tdTomato-positive expression and membrane of mouse liver, spleen, and lung cells. The red color represents tdTomato expression with 599-nm fluorescence. The cyan color represents the membrane with 680- nm fluorescence. Scale bar: 10 μm.

Supplementary Movie 15
Description: Light-field images of the nucleus of Jurkat cells with dualsnapshot scheme, showing the throughput of 5,750 cells/sec. Scale bar: 10 μm
