# Supplementary figures and images for: Light-field flow cytometry for high-resolution, volumetric and multiparametric 3D single-cell analysis
Source: Nat Commun. 2024 Mar 4;15:1975. doi: 10.1038/s41467-024-46250-7 (PMC10912605; doi:10.1038/s41467-024-46250-7)

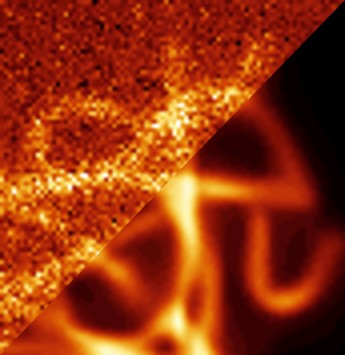

Supplement: Supplementary file 20 — Supplementary Software 1 [file 41467_2024_46250_MOESM20_ESM.zip › Supplementary Software 1/Step4_LFCRecon/acsn.jpg]
